# Dual‐Emission Carbon Dot Nanozymes Mitigate Salt Stress and Enhance Photosynthesis for Crop Germination and Growth

**DOI:** 10.1002/advs.202506906

**Published:** 2025-09-29

**Authors:** Chengfu Su, Xin Peng, Jingying Tan, Yujie Chang, Naifei Pan, Haiying Chen, Xiaokai Xu, Lei Han

**Affiliations:** ^1^ College of Agronomy Qingdao Agricultural University Qingdao 266109 China; ^2^ College of Chemistry and Pharmaceutical Sciences Qingdao Agricultural University Qingdao 266109 China; ^3^ Nano‐Agricultural Engineering Lab Academy of Dongying Efficient Agricultural Technology and Industry on Saline and Alkaline Land in Collaboration with Qingdao Agricultural University Dongying 257347 China; ^4^ National Center of Technology Innovation for Comprehensive Utilization of Saline‐Alkali Land Dongying 257347 China

**Keywords:** carbon dots, crop, dual‐emission, nanozymes, salt stress

## Abstract

Bifunctional carbon dot (CD) nanozymes with dual‐emission properties and superoxide dismutase (SOD)‐like activity are synthesized via microwave method. CDs can simultaneously emit blue and red light under the same excitation wavelength, which effectively overlap with chloroplasts absorption spectra, thereby enhancing energy transfer and photosynthetic efficiency. For hydroponic seedling, CDs significantly promote growth of corn (*Zea mays L*.), increasing fresh weight, shoot length, chlorophyll content, net photosynthetic rate and stomatal conductance by 30.8%, 27.8%, 14.4%, 54.4% and 65.2%, respectively. Furthermore, CDs significantly alleviate oxidative damage in seeds and seedlings through their SOD‐like activity, increasing seed germination rate by 41.5% under NaCl (200 mM) stress. In NaCl (200 mM)‐treated soil, CDs enhanced corn salinity tolerance by triggering coordinated responses including reactive oxygen species scavenging, osmotic adjustment, and metabolic reprogramming. Transcriptome analysis revealed that CDs can regulate key pathways, including energy metabolism, amino acid metabolism, and the synthesis of secondary metabolites. Additionally, by passivating the surface functional groups of CDs, it investigates the specific functional groups contributing to their fluorescence and SOD‐like activity, offering valuable insights for future design on similar nanomaterials. This study provides practical strategies for enhancing crop productivity and resilience in saline‐alkali soils.

## Graphical abstract

Dual‐emission carbon dot nanozymes are synthesized via microwave method. The nanozymes enhance photosynthesis corn seedlings through spectral‐matched light conversion. Their superoxide dismutase‐mimetic activity scavenges salt stress‐induced reactive oxygen species and circumvents energy‐intensive stress responses. Structural‐activity analysis identifies carbonyl‐dominated surface configurations governing this bifunctionality. This study offering practical strategies for enhancing crop productivity and resilience in saline‐alkali soils.

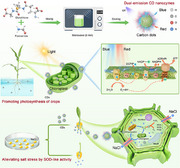

## 1 Introduction

The global population is projected to reach 9.7 billion by 2050,^[^ ^1^ ^]^ intensifying the demand for the sustainable grain production.^[^ ^2^ ^]^ Amidst climate change and limited natural resources, achieving higher food production efficiency with fewer inputs has become a pressing challenge.^[^ ^3^ ^]^ Nanotechnology is recognized as a pivotal tool in the agricultural technological revolution, which offers promising solutions to this challenge.^[^ ^4^ ^]^ Engineered nanomaterials, which are defined as substances with at least one nanoscale dimension (≈1–100 nm),^[^ ^5^ ^]^ can be absorbed by crops through the epidermal stomata, cell walls and root systems, subsequently being transported via the vascular bundles.^[^ ^6^ ^]^ These nanomaterials show significant potential in enhancing crop resistance, improving yield, and enabling precise crop monitoring and management, particularly as protectants, nanofertilizers, and biosensors.^[^ ^7^ ^]^

Currently, a variety of nanomaterials based on metals and metal oxides (such as Ag, CeO_2_, ZnO, CuO, TiO_2_, Mn_3_O_4_, Fe_2_O_3_, and GeO_2_) are widely employed in agriculture as photocatalysts, hormone regulators, antioxidants, and sensing probes.^[^ ^8^ ^]^ However, they may pose potential risks, including environmental pollution, toxicity to crops, and health hazards.^[^ ^9^ ^]^ Although metal nanoparticles exhibit relatively low biotoxicity at low doses, their contact with cells may still trigger significant cellular damage, leading to related pathological changes.^[^ ^10^ ^]^ These potential risks cannot be ignored until their underlying mechanisms are fully understood. Hence, developing nanomaterials with high biocompatibility and environmental safety is essential for building sustainable and high‐quality agricultural systems. These limitations have driven the exploration of carbon‐based nanomaterials as safer alternatives that offer functional effectiveness while reducing ecological risks.^[^ ^11^ ^]^

As a pivotal class of carbon‐based nanomaterials, carbon dots (CDs) have attracted the significant attention since their discovery in 2004, due to their low cost, negligible ecotoxicity, excellent biocompatibility, adjustable photoluminescence, and robust photostability.^[^ ^12^ ^]^ So far, CDs have demonstrated promising applications in biomedicine, environmental protection, photovoltaics, photocatalysts, biosensors and so on,^[^ ^13^ ^]^ with increasing exploration into their agricultural applications.^[^ ^14^ ^]^ Building on these advancements, we are motivated to further explore their potential applications in agricultural in this work. In agriculture, CDs have been reported to promote crop growth and enhance photosynthesis.^[^ ^15^ ^]^ Chloroplasts, which absorb blue and red light, can form hybrid systems with CDs to improve energy transfer to the chloroplasts light‐harvesting centers.^[^ ^16^ ^]^ However, spectrally tailored dual‐emission CDs that precisely match the absorption spectra of chloroplasts (400–500 nm and 650–700 nm) have been scarcely studied.^[^ ^17^ ^]^

Soil salinization has emerged as a critical threat to global agriculture, currently affecting 50% of global arable land and causing continuous soil degradation at three hectares per minute.^[^ ^3^, ^18^ ^]^ This abiotic stress triggers excessive reactive oxygen species (ROS) accumulation in crops, leading to oxidative damage manifested through nucleic acid damage, membrane lipid peroxidation, mitochondrial dysfunction, and homeostasis disorder of Na⁺ and K⁺.^[^ ^8^, ^19^ ^]^ Salinization‐caused ROS overproduction exceeds endogenous superoxide dismutase (SOD) capacity of crops, necessitating exogenous nanozyme supplementation to restore redox homeostasis. Faced with the increasing soil salinization, alleviating salt stress is crucial for sustainable food production. Therefore, it is imperative to develop CDs with SOD‐like activity to assist the antioxidant system in combating abiotic stress and mitigate salinity‐induced yield loss. Although CDs have shown potential in agricultural applications, challenges remain, including limited exploration in enhancing stress tolerance and photosynthesis under high‐salinity conditions.

In this study, we developed dual‐emission CDs with SOD‐like activity via a simple and rapid microwave strategy. Through systematic surface group passivation experiments, we deciphered the structural basis of dual‐emission and SOD‐like activity, revealing a mechanism that provides insight critical for the rational design of multifunctional agricultural nanomaterials. The unique spectral overlap between CD fluorescence and chlorophyll absorption profiles addresses a critical limitation in photosynthetic engineering‐inefficient light utilization beyond the visible spectrum. After a 7‐day treatment with CDs (10 mg L^−1^), hydroponic maize seedlings exhibited significant growth improvements: shoot length, root length, fresh weight and dry weight increased by 27.8%, 12.5%, 30.8%, and 28.5%, respectively. Meanwhile, the SOD‐like activity of CDs (20 mg L^−1^) significantly improved the seed germination of corn (*Zea mays L*.) under NaCl (200 mM) stress by circumventing energy‐intensive stress responses and facilitating morphological recovery, increasing seed germination rate to 86%, which was 41.5% higher than that of the salt‐stress group. Leveraging the dual functionality, CDs enabled corn seedlings to achieve growth performance statistically equivalent to that of non‐stressed controls under simulated high‐salinity conditions (200 mM NaCl). In NaCl (200 mM)‐treated soil, CDs enhanced corn salinity tolerance by triggering coordinated responses, including ROS scavenging, osmotic adjustment, and metabolic reprogramming. Transcriptome analysis further revealed that CDs treatment activated key metabolic pathways associated with salt‐tolerance, offering valuable molecular insights into the mechanisms behind CD‐enhanced salt stress tolerance in corn. These findings establish CDs as a powerful tool simultaneously addressing photosynthetic limitation and oxidative damage in saline agriculture, providing a blueprint for sustainable crop resilience enhancement.

## 2 Results and Discussion

### 2.1 Preparation and Characterization of CDs

**Scheme 1:**
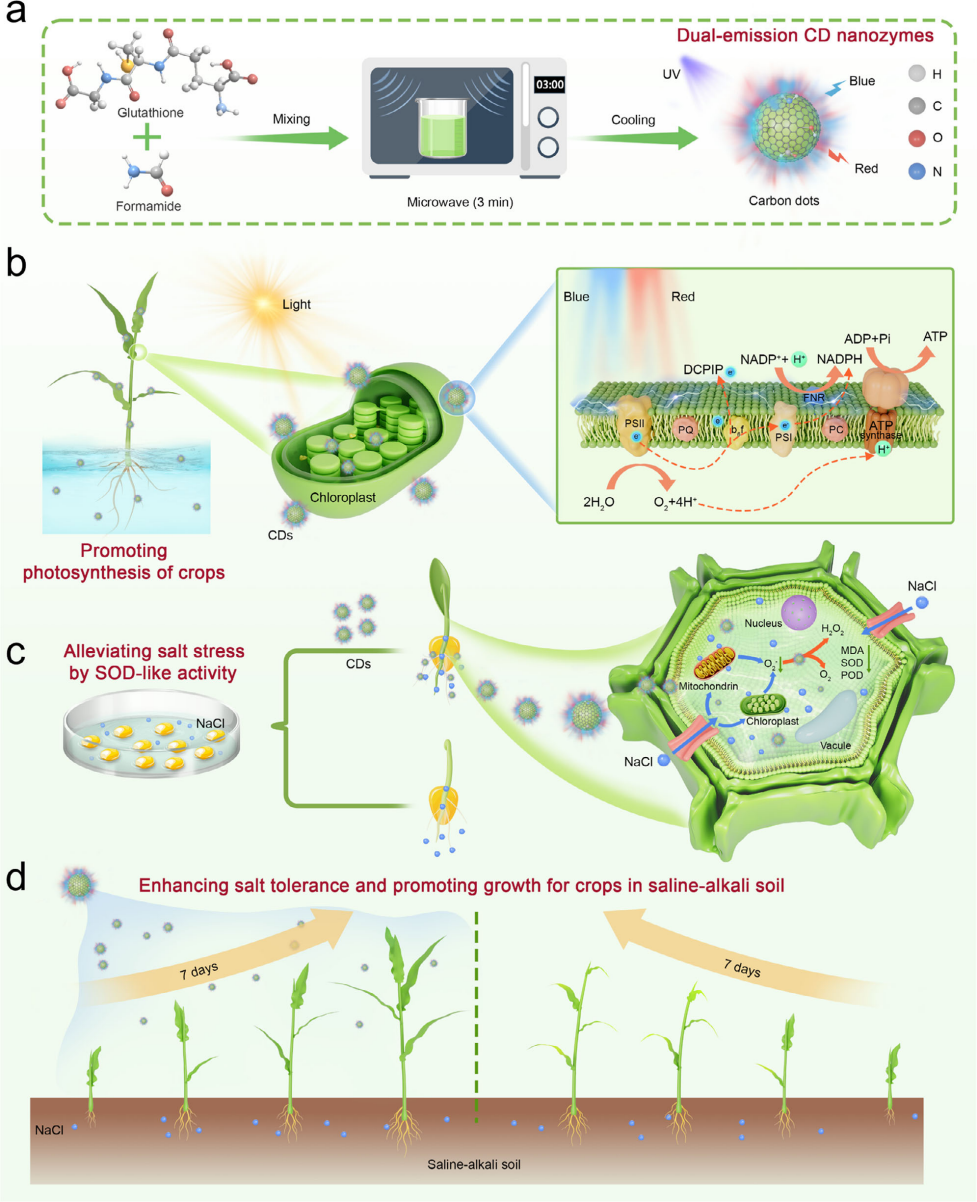
Proposed experimental design. a) Microwave‐assisted synthesis dual‐emission CD nanozymes from glutathione/formamide precursors. b) Photosynthetic enhancement via spectral‐matched fluorescence and electron transport facilitation in chloroplasts. c) Salt stress mitigation of corn seeds through SOD‐like activity. d) Synergetic growth promotion in saline soils through photonic and antioxidant functions of CD nanozymes.

**Figure 1:**
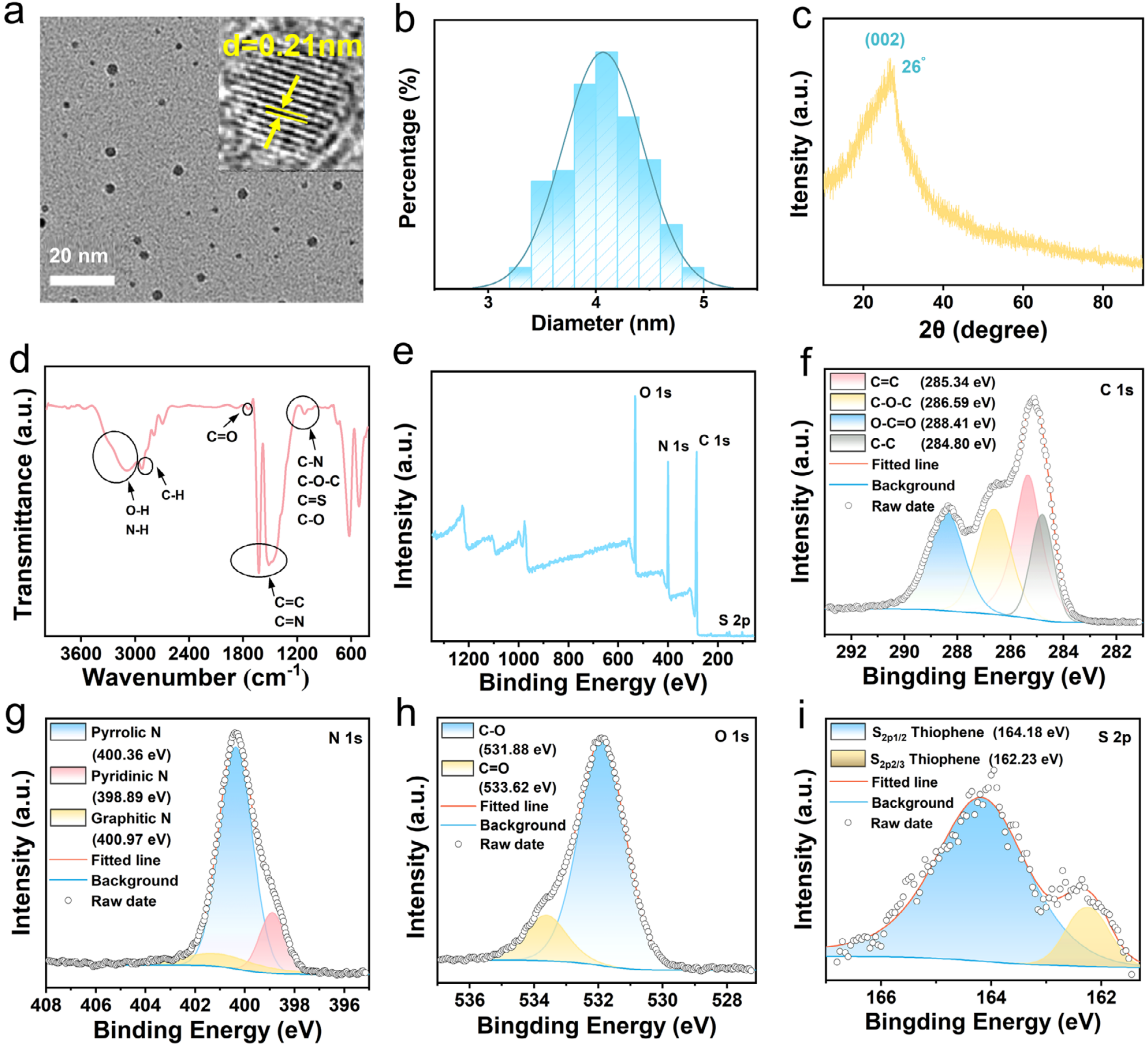
Characterization of CDs structure. a) TEM image of CDs (Inset: high resolution TEM). b) Particle size distribution of CDs. c) XRD pattern of CDs. d) FTIR spectrum of CDs. e) XPS spectra of CDs and f−i) high‐resolution C 1s, N 1s, O 1s, and S 2p spectra.

The synthesis and agricultural application of the multifunctional CDs were illustrated in Scheme 1a. The CDs were synthesized by microwave heating of solution containing glutathione and formamide. The resulting solution was dialyzed and subsequently vacuum‐freeze‐dried to obtain a powder (Figure S1, Supporting Information). The transmission electron microscopy (TEM) of the synthesized CDs revealed their excellent dispersibility and spherical morphology. The lattice spacing of 0.21 nm corresponded to the (100) plane of graphene (**Figure** **1**a). Statistical analysis of over 100 particles determined the average particle size to be 4.2 nm (Figure 1b). X‐ray diffraction (XRD) analysis showed a peak at 26°, corresponding to the (002) plane of graphene (Figure 1c).

Further structural and compositional analysis was performed using Fourier transform infrared (FTIR) and X‐ray photoelectron spectroscopy (XPS). The FTIR spectrum revealed a variety of functional groups on the surface of the CDs. The broad absorption band at 3000–3500 cm^−1^ was attributed to the stretching vibrations of –OH and –NH groups. Absorption peaks at 2925 cm^−1^1 corresponded to the stretching vibration of aliphatic C─H bonds. Additionally, the bands at 1625 cm^−1^ and 1514 cm^−1^ were associated with C═C and C═N stretching vibrations, while the bands between 1000 and 1230 cm^−1^ were attributed to C─N, C─O─C, C═S, and C─O bonds (Figure 1d).

XPS analysis confirmed the presence of carbon (C 1s), nitrogen (N 1s), oxygen (O 1s), and sulfur (S 2p) in the CDs, with binding energies of 285, 400, 531, and 164 eV, respectively (Figure 1e). High‐resolution XPS spectra of C 1s, N 1s, O 1s, and S 2p were fitted to determine the chemical states of these elements. The C 1s spectrum (Figure 1f) showed peaks at 285.34, 286.59, 288.41, and 284.80 eV, corresponding to C═C, C–O–C, C═O, and C–C, respectively. For the N 1s spectrum (Figure 1g), three peaks at 400.36, 398.89, and 400.97 eV were assigned to pyrrole nitrogen, pyridine nitrogen and graphite nitrogen, respectively. The O 1s spectrum (Figure 1h) exhibited two peaks at 531.6 and 533.88 eV, attributing to C–O and C═O groups. Finally, the S 2p spectrum (Figure 1i) revealed two peaks at 164.2 and 162.3 eV, confirming the presence of thiophene‐derived sulfur species (S 2p_3_/_2_ and S 2p_1_/_2_).

### 2.2 Dual‐Emissive CDs with Surface Group‐Engineered Fluorescence

**Figure 2:**
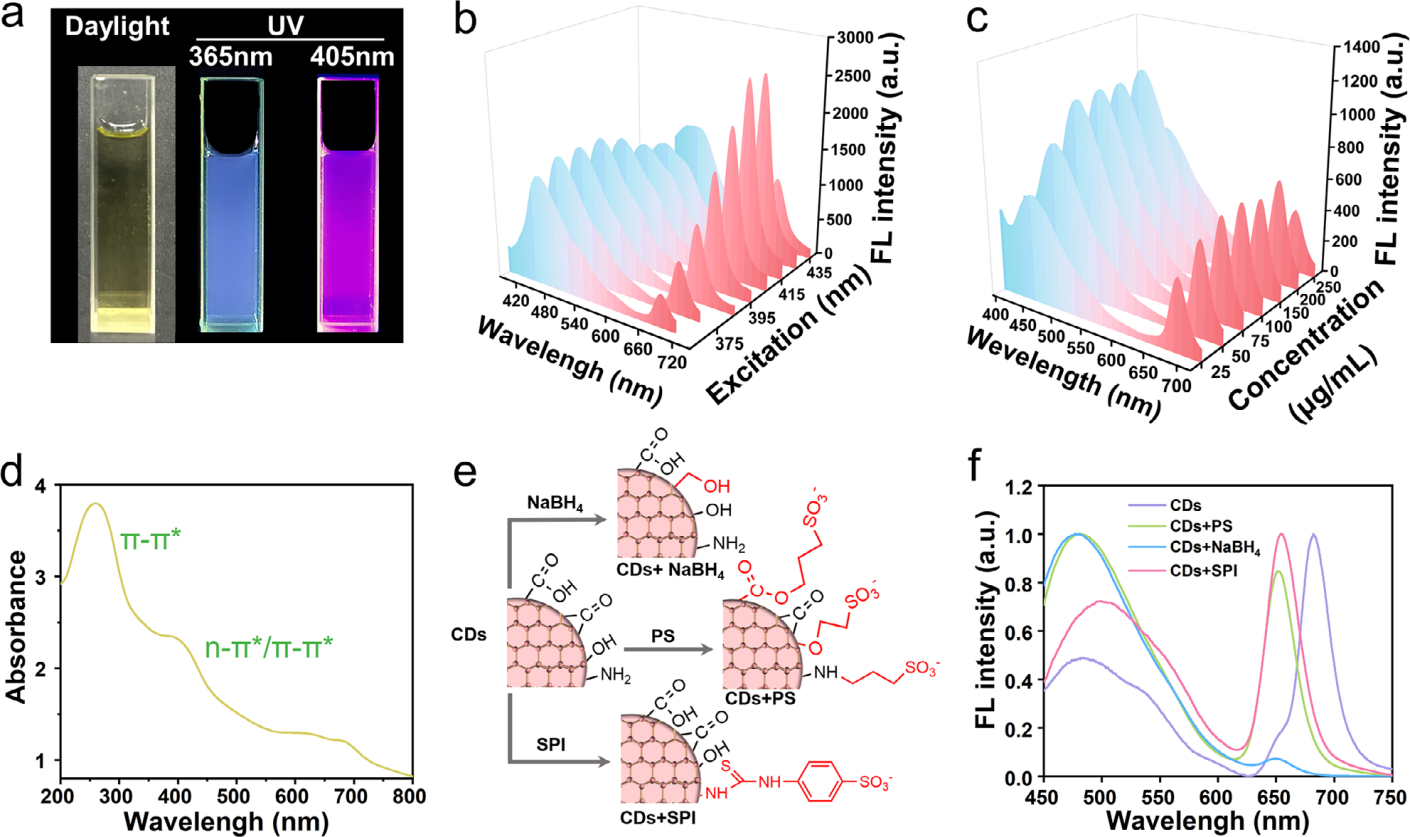
Optical properties of CDs. a) Photographs of aqueous solutions of CDs under daylight and UV light (365 and 405 nm). b) Emission spectra of aqueous solutions of CDs at different excitation wavelengths. c) Emission spectra of different concentrations of CDs excited at 375 nm. d) UV–vis absorption spectrum of CDs. e) Illustration of surface group modification. f) Fluorescence spectra of modified CDs under 415 nm excitation.

The optical properties of the synthesized CDs were investigated. The aqueous solution of CDs showed yellow‐green color under daylight. When exposed to UV light at 365 and 405 nm, CDs solution displayed apparent blue and red fluorescence, respectively (**Figure** **2**a). The fluorescence intensities of both the blue (466 nm) and red (683 nm) emissions peaked when excited at 395 and 415 nm, respectively (Figure 2b). As the concentration of CDs increased from 25 to 200 µg mL^−1^, the fluorescence intensity gradually increased (Figure 2c). However, at higher concentrations, aggregation‐induced quenching was observed. The chemical bonds of CDs were further determined through the UV–vis absorption spectroscopy (Figure 2d). Three absorption peaks were observed at 260 nm, 380–420 nm and 600–700 nm, corresponding to the π→π* transition of the aromatic C═C bond, as well as the π→π* and n→π* transitions in the aromatic π system containing C═O, C═N, and C═S bonds, respectively,^[^ ^20^ ^]^ confirming the coexistence of aromatic carbon cores and heteroatom‐rich surface moieties.

Based on the structural composition of CDs, which typically consist of sp^2^ carbon core and surface domains,^[^ ^21^ ^]^ we hypothesized that the observed dual‐emission behavior of CDs originated from these two distinct domains.^[^ ^22^ ^]^ To verify this hypothesis, we performed selective surface group passivation using three chemical reagents: 1,3‐propanesulfonate (PS), sodium borohydride (NaBH_4_), 4‐sulfophenyl isothiocyanate sodium salt (SPI) (Figure 2e). As demonstrated in Figure 2f, the fluorescence emission of CDs could be changed by altering surface functional groups. PS could form ether with hydroxyl groups, ester with carboxyl groups, and secondary amines with amino groups.^[^ ^23^ ^]^ The fluorescence spectrum of CDs + PS showed no change in the position of first fluorescence peak, but the second peak shifted from 683 to 652 nm, compared with CDs. SPI could react with amino groups to form isothiourea bonds, showing minimal effects on the emission profile.^[^ ^24^ ^]^ NaBH_4_ could reduce carbonyl groups to hydroxyl groups. After the reduction, the blue emission peak of CDs + NaBH_4_ redshifted from 481 to 499 nm, and the red emission peak blueshifted from 683 to 652 nm. It was worth noting that the red emission peak of CDs + NaBH_4_ almost disappeared under 415 nm excitation, suggesting that carbonyl groups played a vital role in regulating the red fluorescence emissions of CDs. Furthermore, when excited at different wavelengths (365–435 nm), the dual‐emission behavior was preserved for CDs + PS and CDs + SPI, but the red peak for CDs + NaBH_4_ was virtually absent (Figure S2, Supporting Information). Therefore, these findings clearly demonstrate that surface functional groups, particularly carbonyl groups, play a vital role in regulating the red fluorescence emission of CDs, while the blue emission likely originates from the intrinsic carbon core structure.

### 2.3 Evaluation of Promoting Photosynthesis in Vitro

**Figure 3:**
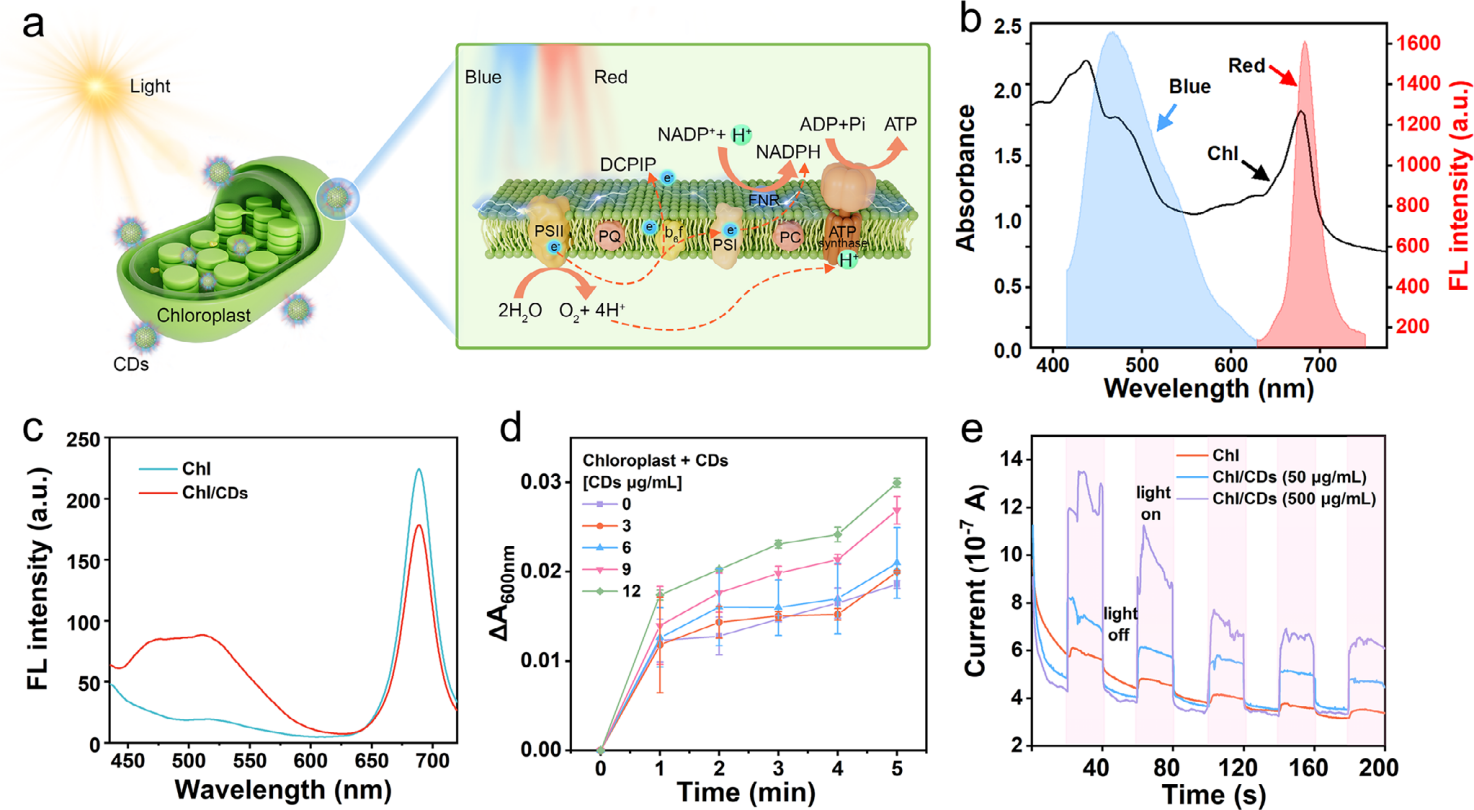
In vitro characterization of CD‐enhanced photosynthetic activity. a) Schematic illustration of the proposed mechanism for CD‐mediated photosynthetic enhancement. b) Absorption spectrum of chloroplasts (Chl), and fluorescence emission spectrum of CDs excited at 395 nm in water. c) Fluorescence emission spectra of native chloroplasts, and Chl/CDs complex, excited at 375 nm. d) DCPIP reduction kinetics mediated by Chl/CDs complexes with varying concentrations of CDs (0–12 µg mL^−1^) under the light illumination (4 mW cm^−2^). Error bars: mean ± SD (n=3). e) Photocurrent response of Chl with/without CDs (50 and 500 µg mL^−1^) under 365 nm UV light irradiation (20 s on/off cycles).

The core of photosynthesis in the thylakoid membrane comprises four key components: photosystem II (PSII), cytochrome b6f complex, photosystem I (PSI), and adenosine triphosphate (ATP) synthase, which collectively drive light‐dependent reactions.^[^ ^25^ ^]^ PSII pigment‐protein complexes capture a part of the solar spectrum to initiate water photolysis, generating oxygen, protons and electrons.^[^ ^26^ ^]^ Protons and electrons are transferred to PSI though a proton gradient and an electron transport chain, and store as stable chemical energy in ATP and nicotinamide adenine dinucleotide phosphate (NADPH), respectively (**Figure** **3**a).^[^ ^26a^ ^]^ However, photosynthesis mainly utilizes the visible light spectrum within the range of 400–700 nm, which accounts for 48.7% of the incident solar energy,^[^ ^27^ ^]^ highlighting the critical need for strategies to expand light absorption wavelength of chloroplasts in crops.

Our synthesized CDs address the above limitation through unique dual‐emission fluorescence that overlayed with chloroplasts absorption spectra (Figure 3b). Following the mixing of CDs with chloroplasts, the centrifuged precipitate of chloroplasts exhibited not only the inherent red fluorescence peak of chlorophyll at 680 nm, but also an additional blue emission, confirming the successful integration of chloroplast/CDs complex (Chl/CDs) (Figure 3c).

To quantify photosynthetic enhancement, we employed the Hill reaction assay monitoring 2,6‐dichlorophenol‐indophenol (DCPIP) reduction kinetics. The Chl/CDs complex demonstrated obviously faster DCPIP decolorization compared to native chloroplasts (Figure 3d; Figure S3, Supporting Information). Further, photoelectrochemical measurements provided complementary evidence by measuring the cyclic photocurrent of chloroplasts and Chl/CDs complex under light stimulation. The photocurrent response of individual chloroplasts was weak, but CD incorporation significantly boosted the photocurrent generation of chloroplast (Figure 3e). The enhancement of chloroplast photocurrent likely originates from improved charge separation efficiency and accelerated electron transfer rates by CD in chloroplasts (Scheme 1b).^[^ ^28^ ^]^ The above results provided effective evidence for the function of CDs to promote the photosynthetic activity of chloroplasts.

### 2.4 Promoting Photosynthesis and Growth of Crops

**Figure 4:**
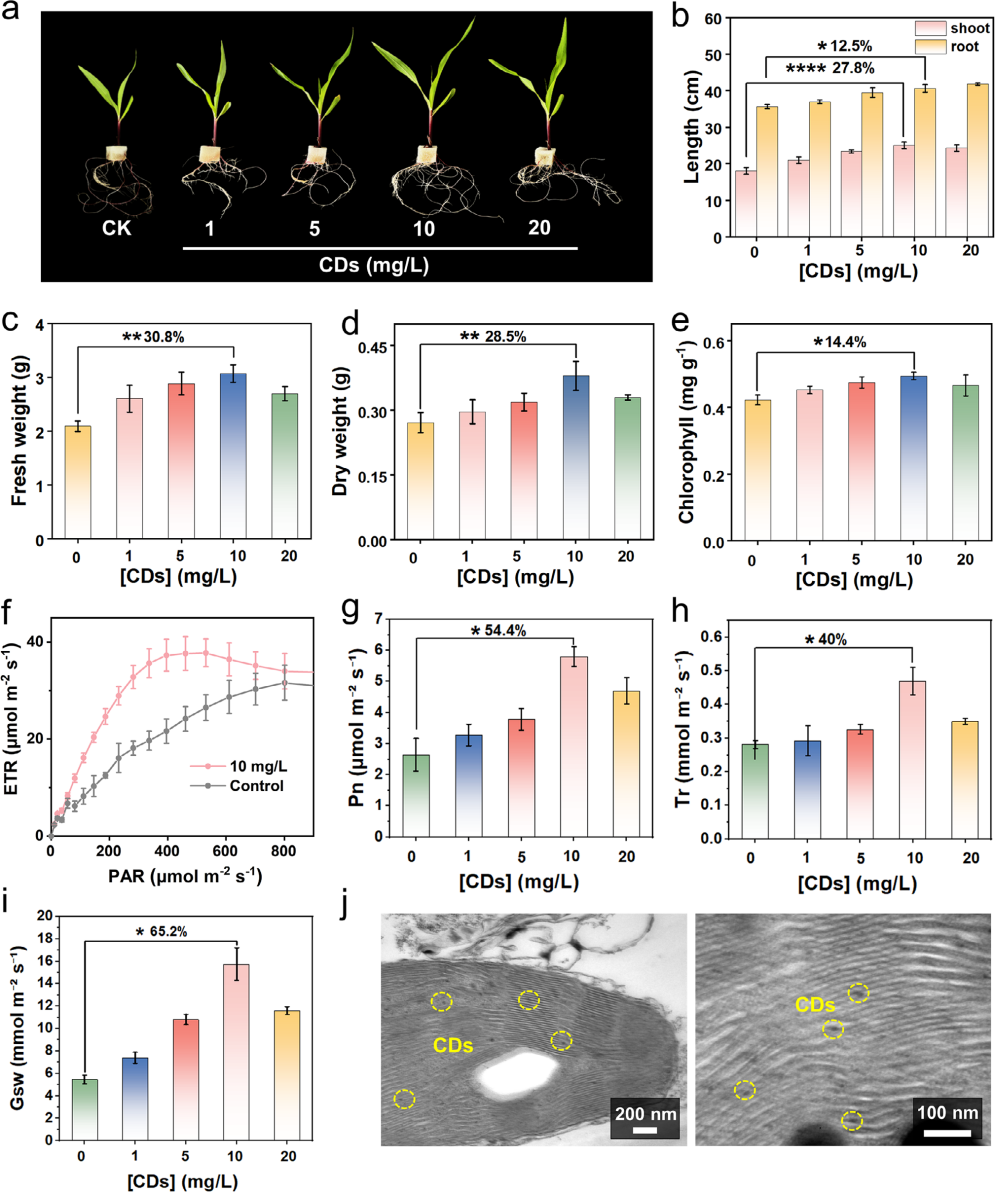
CDs‐mediated enhancement of photosynthetic performance and growth in hydroponic corn seedlings. a) Phenotypic comparison of corn seedlings treated with different concentrations of CDs (1–20 mg L^−1^) after 1 week of hydroponic culture. The corresponding quantitative analysis of b) the length of shoot and root, c) fresh weight, d) dry weight and e) chlorophyll content. f) ETR of corn seedlings treated with and without 10 mg L^−1^ CDs. g–i) Gas exchange parameters of corn seedlings under different CDs concentrations: (g) Pn, (h) Tr and (i) Gsw. j) TEM images of showing CDs localization in corn leaf tissue. Error bars: mean ± SD (n=3). Statistical significance was determined by one‐way ANOVA with Tukey's test. *p < 0.05; **p < 0.01; ****p < 0.0001; ns, not significant.

Building on the demonstrated potential of CDs to enhance photosynthetic activity, we systematically evaluated their effects on crop growth using corn seedlings as a model (**Scheme** **1b**). When the seedlings grew to two‐leaf‐one‐heart stage, they were treated with different concentrations of CDs by hydroponics for one week. Optimal growth enhancement was observed at 10 mg L^−1^ of CD treatment (**Figure** **4**a), with significant improvements in multiple growth parameters. Specifically, shoot and root length increased by 27.8% and 12.5%, respectively (Figure 4b). The fresh weight and dry biomass increased by 30.8%, and 28.5% (Figure 4c,d). A 14.4% increase in chlorophyll content (Figure 4e), and an increase in electron transport rates (ETR) (Figure 4f) collectively indicated the improved photosystem II efficiency. To further support this, we measured the key gas exchange parameters, including net photosynthetic rate (Pn), stomatal conductance (Gsw), transpiration rate (Tr), and instantaneous water use efficiency (IWUE). As shown in Figure 4g‒I, and Figure S4 (Supporting Information), treatment with CDs (10 mg L^−1^) significantly enhanced these parameters, with increases of 54.4%, 65.2%, 40%, and 24.5%, respectively. These coordinated enhancements demonstrate that CDs can effectively promote photosynthetic efficiency, through enhanced chloroplast activity and optimized stomatal behavior, thereby promoting corn seedling development. TEM further confirmed the subcellular localization of CDs near chloroplasts (Figure 4j), providing direct evidence for their close association with photosynthetic machinery.

**Figure 5:**
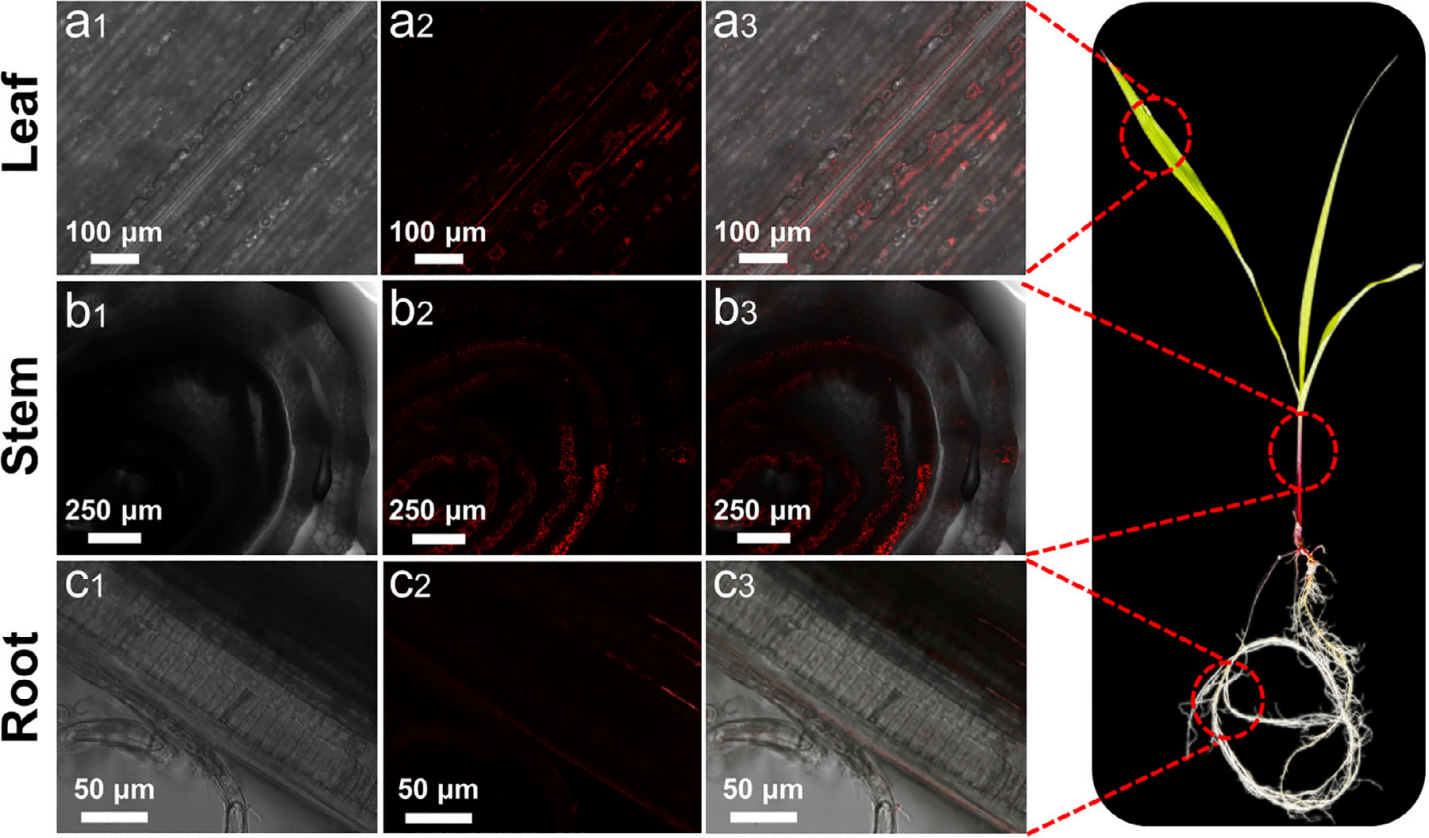
CLSM images of CDs distribution in corn seedlings treated with CDs solution (10 mg mL^−1^) for 7 days under 405 nm excitation. The data of leaf tissue a), cross section of stem b), and longitudinal section of root c) were collected in bright field (1), red emission (630−700 nm) (2), and the overlay of fluorescence and bright field images (3).

Previous studies have demonstrated that nanoparticles can penetrate the crops through cell walls or stomata, and be transported to various tissues by vascular bundles.^[^ ^29^ ^]^ Therefore, to observe the uptake and transport of CDs in corn seedlings, we employed confocal laser scanning microscopy (CLSM) to track CD distribution in different plant tissues. The seedlings were cultured in 1/2 Hoagland nutrient solution containing CDs (10 mg L^−1^) for 7 days, followed by thorough washing with deionized water to remove surface‐adsorbed nanoparticles. CLSM imaging revealed strong red fluorescence signals in roots, stems, and leaves of CDs‐treated plants (**Figure** **5**a–c), while control plant showed minimal autofluorescence (Figure S5, Supporting Information). The spatial distribution pattern indicated that CDs were absorbed through root tissues, and transported upward along the vascular bundles, ultimately accumulating in leaf mesophyll cells. These findings collectively demonstrate that CDs can effectively penetrate plant tissues, exhibit excellent biocompatibility, and exert their light‐conversion properties to promote crop growth.

### 2.5 SOD‐Like Activity and Antioxidant Capability of CDs

**Figure 6:**
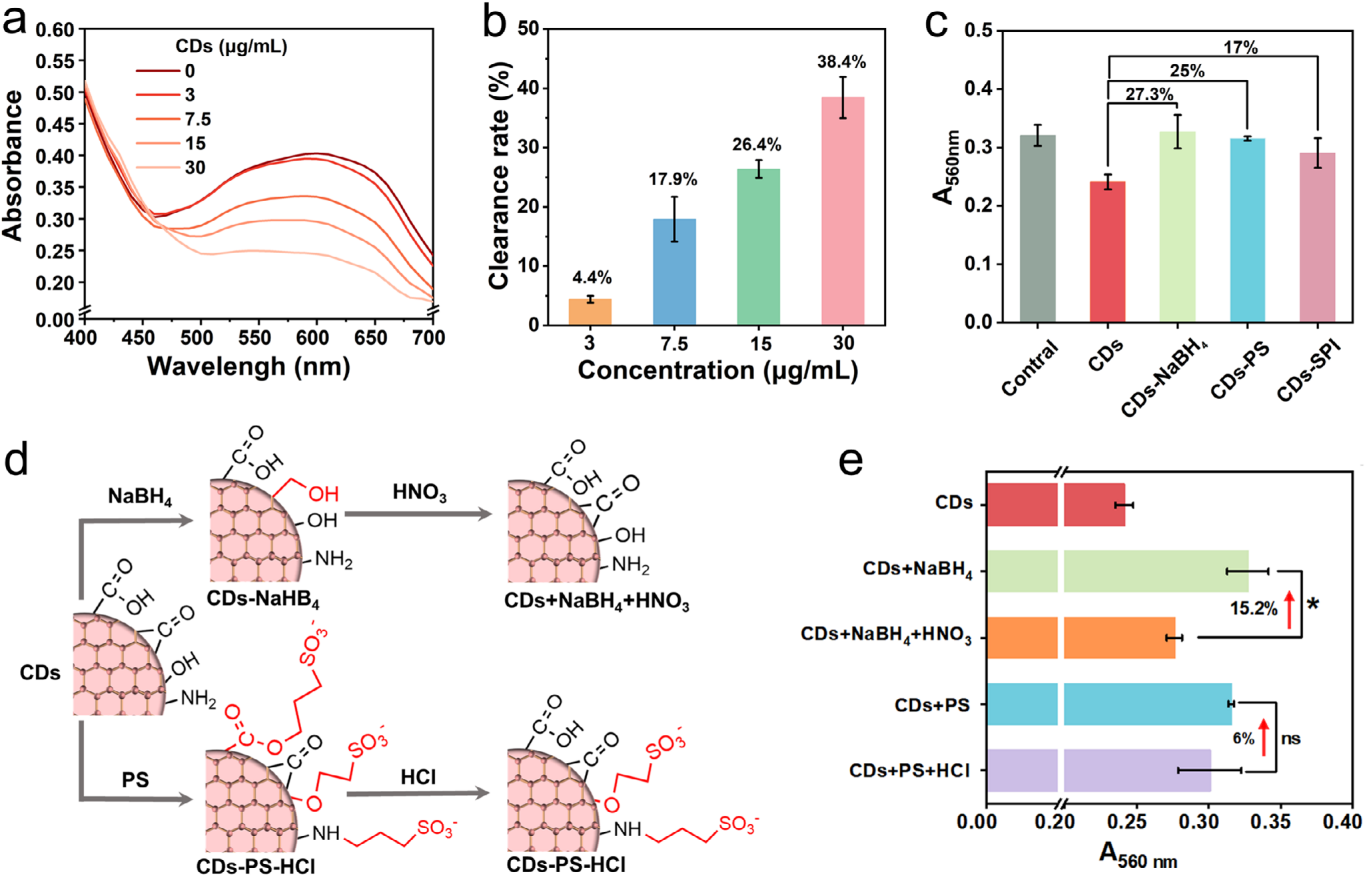
Characterization of CDs antioxidant performance. a) NBT reduction assay for SOD‐like activity of different concentration of CDs. b) O_2_·^−^ scavenging efficiency at different CDs concentrations. c) SOD‐like activity comparison of surface‐modified CDs: CDs + NaBH_4,_ CDs + PS and CDs + SPI. d) Illustration of modifications of carbonyl, carboxyl, and hydroxyl groups on the surface of CDs. e) Quantitative changes in SOD‐like activity after the recovery of passivated surface groups of CDs. Error bars: mean ± SD (n=3). Statistical significance was determined by one‐way ANOVA with Tukey's test. *p < 0.05; ns, not significant.

The SOD‐mimetic activity of CDs was first verified through the nitroblue tetrazolium (NBT) reduction assay. The superoxide anion (O_2_·^−^), a key ROS in biological systems, was generated by the photolysis of riboflavin, and its concentration could be quantified by NBT, where O_2_·^−^ converted NBT to blue formazan. With the increase of CDs concentration, the absorption value of NBT at 560 nm gradually decreased (**Figure** **6**a), indicating the scavenging capability of CDs to O_2_·^−^. At 30 µg mL^−1^, CDs could scavenge ≈38.4% of O_2_·^−^ (Figure 6b), showed excellent SOD‐like activity and antioxidant capability of CDs.

Some reports have indicated that the antioxidant functionality of CD nanozymes primarily stems from the electronic transfer, and hydrogen atom transfer of surface functional groups.^[^ ^9^, ^30^ ^]^ Our previous findings (Figure 1d–i) revealed that the surface of CDs were decorated with diverse functional groups including carboxyl, carbonyl, hydroxyl and amino groups. So, to elucidate the contribution of specific functional groups to SOD‐like activity, we conducted systematic modifications and passivation for surface groups of CDs: 1) carbonyl group reduction using NaBH_4_ (CDs + NaBH_4_), 2) hydroxyl and carboxyl group passivation via PS modification (CDs + PS), and 3) amino group passivation through SPI (CDs + SPI). These modifications resulted in 27.3%, 25%, and 17% reductions in SOD‐like activity, respectively (Figure 6c), demonstrating the collective contribution of these functional groups to SOD‐like activity.

To further validate these findings, we performed functional group regeneration experiments (Figure 6d). Oxidation of CDs + NaBH_4_ with HNO_3_ restored 15.2% of SOD‐like activity by regenerating carbonyl groups, while hydrolysis of CDs + PS with HCl recovered only 6% activity due to partial restoration of carboxyl groups (Figure 6e). These results confirm that carbonyl groups might serve as crucial role, while carboxyl, amino and hydroxyl groups provide additional effect for SOD‐like activity and antioxidant capacity.

The total antioxidant capacity was further evaluated using 2,2′‐azinobis‐(3‐ethylbenzthiazoline‐6‐sulphonate) cation radical (ABTS^·+^). CDs demonstrated concentration‐dependent ABTS^·+^ clearing effect, and the clearance rate of ABTS^·+^ reached 93% at 66 µg mL^−1^ (Figure S6, Supporting Information) The reduction of ABTS^·+^ maybe was due to hydrogen donation and electron transfer mechanisms of CDs’ surface groups.^[^ ^9^, ^31^ ^]^ These comprehensive analyses establish CDs as potent antioxidants with significant potential for mitigating oxidative stress in biological systems.

### 2.6 Mitigation of Salt Stress‐Induced Oxidative Damage of Corn Seeds

**Figure 7:**
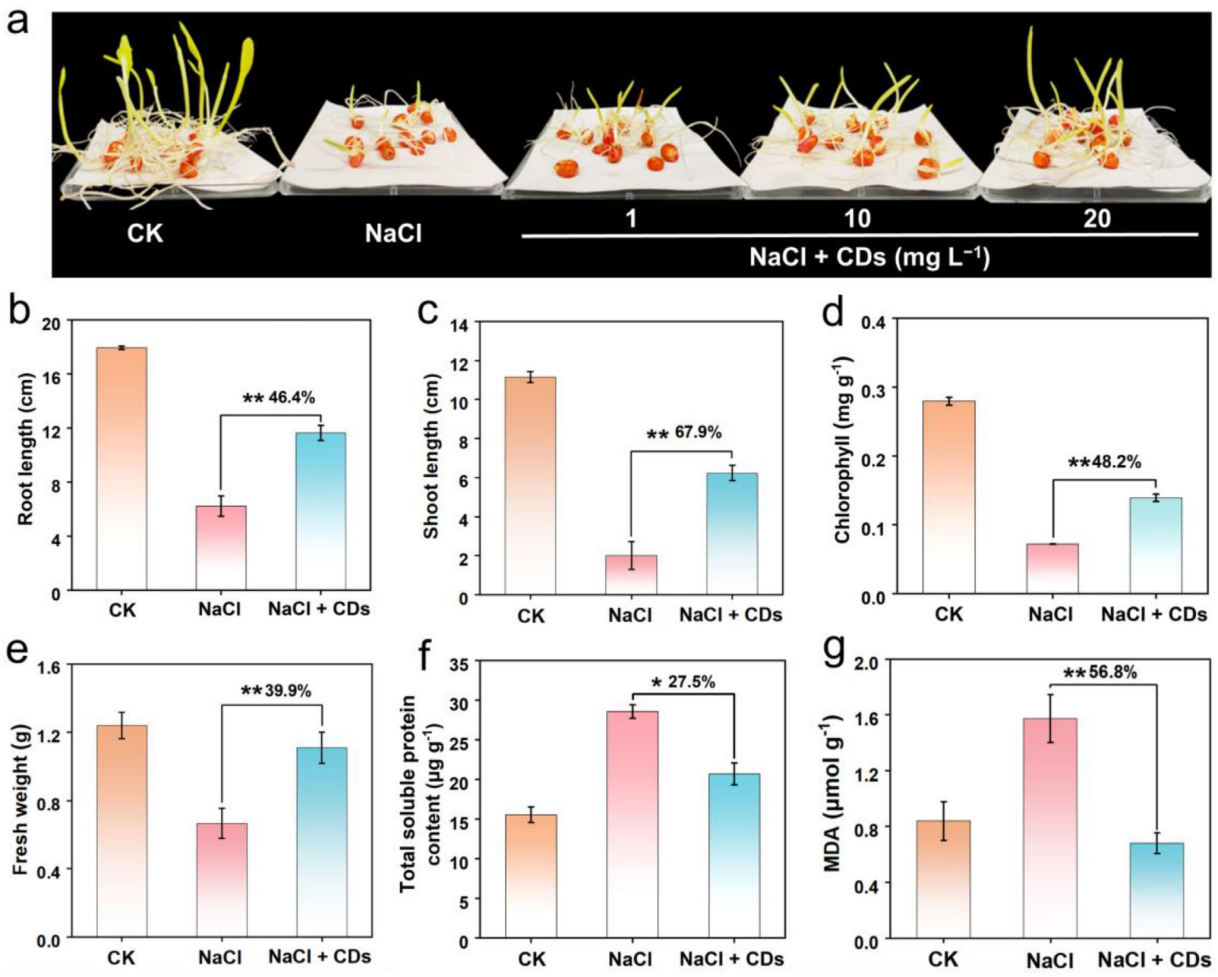
CDs‐mediated protection of corn seeds under salt stress. a) Germination phenotypes with CDs treatments (1, 10, and 20 mg L^−1^) under NaCl stress (200 mM) for 1 week. Quantitative analysis of b) root length, c) shoot length, d) chlorophyll content, e) fresh weight, f) total soluble protein content and g) MDA content in corn seeds under salt stress with and without CDs treatment (20 mg L^−1^). Error bars: mean ± SD (n=3). Statistical significance was determined by one‐way ANOVA with Tukey's test. *p < 0.05; **p < 0.01.

Prior to the plant experiments, we measured the hydrodynamic size distribution and zeta potential of CDs in various solutions, including H_2_O, NaCl solution (200 mM) and 1/2 Hoagland nutrient solution (Figure S7, Supporting Information). The average hydrodynamic diameters of CDs in these solutions were 750.7 nm, 455 nm, and 563.3 nm, respectively and the zeta potentials of CDs in the three systems were all negative, suggesting minimal solution‐dependent effects on CDs. Salt stress can induce the excessive accumulation of ROS in crops, inhibiting their growth and development.^[^ ^32^ ^]^ Inspired by their SOD‐like and antioxidant activities of CDs, we investigated their protective effects against salt stress during seed germination (Scheme 1c). The disinfectant corn seeds were placed in germinating box containing NaCl (200 mM) with or without CDs for 7 and 14 days. Exposure to NaCl significantly impaired corn seed germination, while CDs pretreatment remarkably alleviated this damage (**Figure** **7**a; Figure S9a, Supporting Information). After 7 days of seed germination, the germination rate of the NaCl‐treated group was only 50.3%, which was significantly lower than that of the control group. After treatment with CDs (20 mg L^−1^) for 7 days, the germination rate was restored to 86% (Figure S9b, Supporting Information), which was 41.5% higher than that of the salt‐stress group, fully indicating that CDs have a good promoting effect on the seed germination under salt stress.

Comprehensive physiological analysis for 7 days of seed germination revealed CDs‐mediated stress mitigation through multiple pathways (Figure 7b–g). As for morphological recovery, CDs‐treated seeds exhibited 46.4% and 67.9% increases in root and shoot lengths compared to salt‐stressed controls (Figure 7b,c). Salt stress exerts an inhibitory effect on PSII, and excessive ROS production impairs chlorophyll biosynthesis.^[^ ^33^ ^]^ The 48.2% chlorophyll content recovery (Figure 7d) correlated with reduced PSII photoinhibition, demonstrating CDs‐mediated photosynthetic protection. This chlorophyll restoration was supported by a 39.9% increase in fresh weight (Figure 7e), reflecting improved water status essential for chlorophyll biosynthesis.^[^ ^28^ ^]^ As for the metabolic level, CD treatment reduced soluble protein content by 27.5% (Figure 7f), suggesting restored osmotic balance. Malondialdehyde (MDA) is a toxic product of lipid peroxidation under oxidative stress. 56.8% decrease in MDA content (Figure 7g) confirmed reduced membrane lipid peroxidation. The excessive accumulation of ROS causes an upregulation of antioxidant enzymes (SOD and peroxidase (POD)) in the seeds. CDs treatment decreased these enzymes (Figure S8, Supporting Information), indicating direct radical scavenging rather than enzymatic induction, thereby preserving metabolic resources for growth processes.

To further explore the protective effect of CDs on corn seeds, we extended the germination period to 14 days under salt stress conditions and evaluated key growth parameters (Figure S9c‒h, Supporting Information). After treatment with CDs, the fresh weight, dry weight, root length, and shoot length of corn seedlings increased by 17%, 20.4%, 38.7%, and 36.2%, respectively, compared with the NaCl‐treated group (Figure S9c‒f, Supporting Information). Moreover, the content of O_2_·^−^ and H_2_O_2_ decreased by 18.6% (Figure S9, Supporting Information) and 15.4%, respectively (Figure S9g,h, Supporting Information), demonstrating the antioxidant effect of CDs on corn seedlings. Taken together, these above results suggest that CDs penetrate seeds and exert protective effects through a nanozyme‐mediated antioxidant mechanism, facilitating morphological recovery, photosynthetic protection, metabolic homeostasis, oxidative stress reduction, and efficient resource allocation (Scheme 1c).

### 2.7 Dual‐Functional Enhancement of Seedling Performance under Salinity Stress

**Figure 8:**
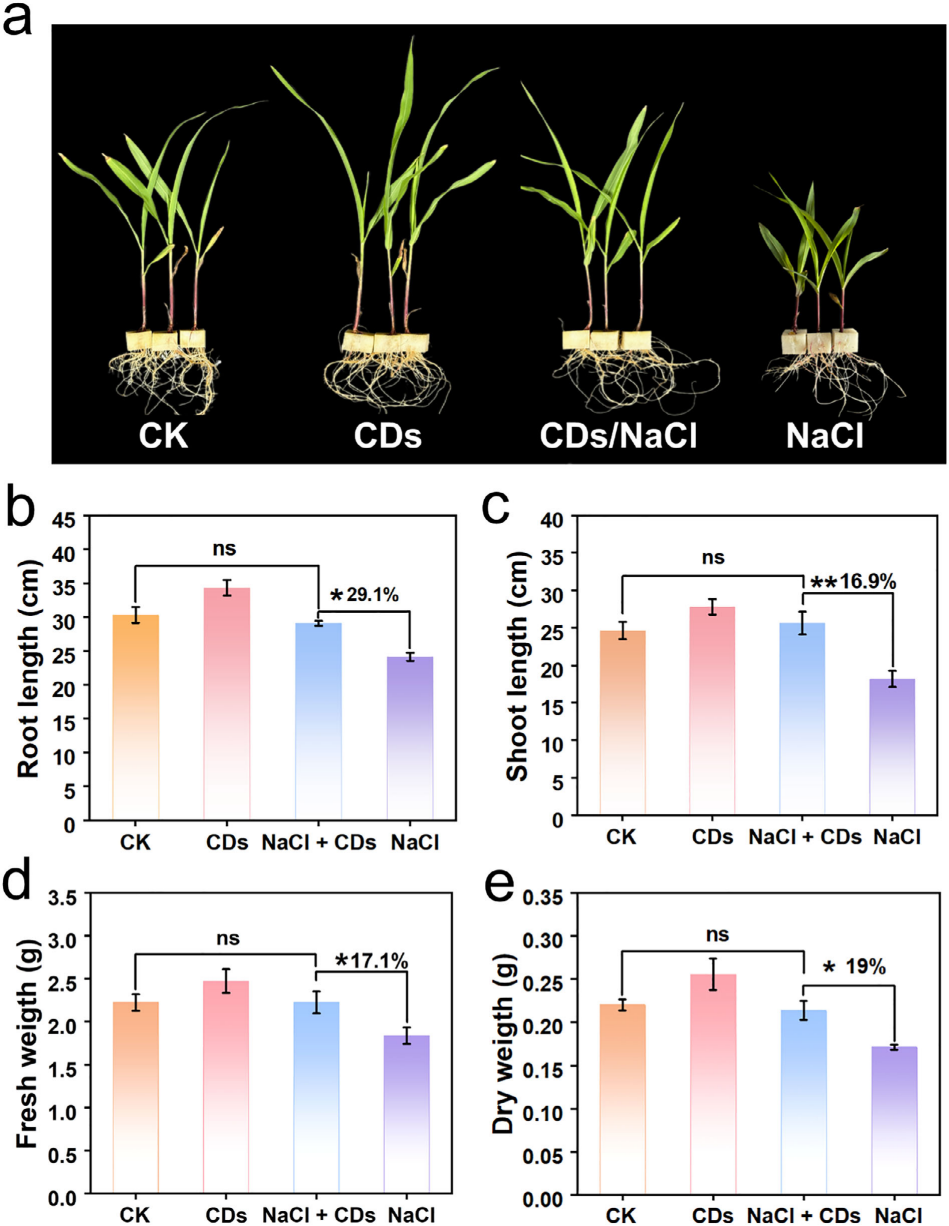
Effects of CDs on the growth of corn seedlings under highly saline conditions. a) Representative images of seedlings subjected to four different treatments (CK, CDs, NaCl + CDs, NaCl) for one week. Quantitative growth parameters: b) shoot length, c) root length, d) fresh weight, and e) dry weight. Error bars: mean ± SD (n=3). Statistical significance was determined by one‐way ANOVA with Tukey's test. *p < 0.05; **p < 0.01; ns, not significant.

The synergistic effects of CDs‐mediated photosynthetic enhancement and antioxidant protection were systematically evaluated under simulated saline conditions using two‐leaf‐stage corn seedlings as models. Seedlings were sprayed daily with distilled water or equivalent volumes of CDs aqueous solution (20 mg L^−1^). Under non‐saline conditions, seedlings treated with CDs for 7 days exhibited superior growth performance compared to water‐sprayed controls, confirming the photosynthetic‐promoting effects of CDs (**Figure** **8**a). Corn seedlings subjected to NaCl (200 mM) stress exhibited severe growth inhibition, which was effectively mitigated by CDs treatment (Figure 8a). Compared to salt‐stressed controls, CDs‐supplemented seedlings showed 29.1% and 16.9% increases in root and shoot lengths, respectively (Figure 8b,c), along with 17.1% and 19% improvements in fresh and dry weights (Figure 8d,e). Remarkably, these growth parameters of NaCl + CDs group matched those of non‐stressed control (CK) (Figure 8a–e), confirming the dual‐functional efficacy of CDs enables seedlings to grow at a normal level under 200 mM salt stress.

The robust stress mitigation and resilience of CDs‐treated seedlings under high salinity likely stem from the coordinated action of CDs‐enabled mechanisms: (1) Enhanced photosynthetic electron transport and improved light‐energy utilization ensured sustained energy production; (2) SOD‐like activity minimized ROS‐induced cellular damage; (3) CDs prevented the accumulation of stress‐induced metabolites. By circumventing energy‐intensive stress responses, CDs redirected metabolic resources toward growth processes, achieving salt stress mitigation without compromising developmental vigor. This integrated functionality positions CDs as a promising nano‐enabled strategy for sustainable crop cultivation in saline‐alkaline soils.

### 2.8 Integrated Assessment of Seedlings in Soil under Salt Stress

**Figure 9:**
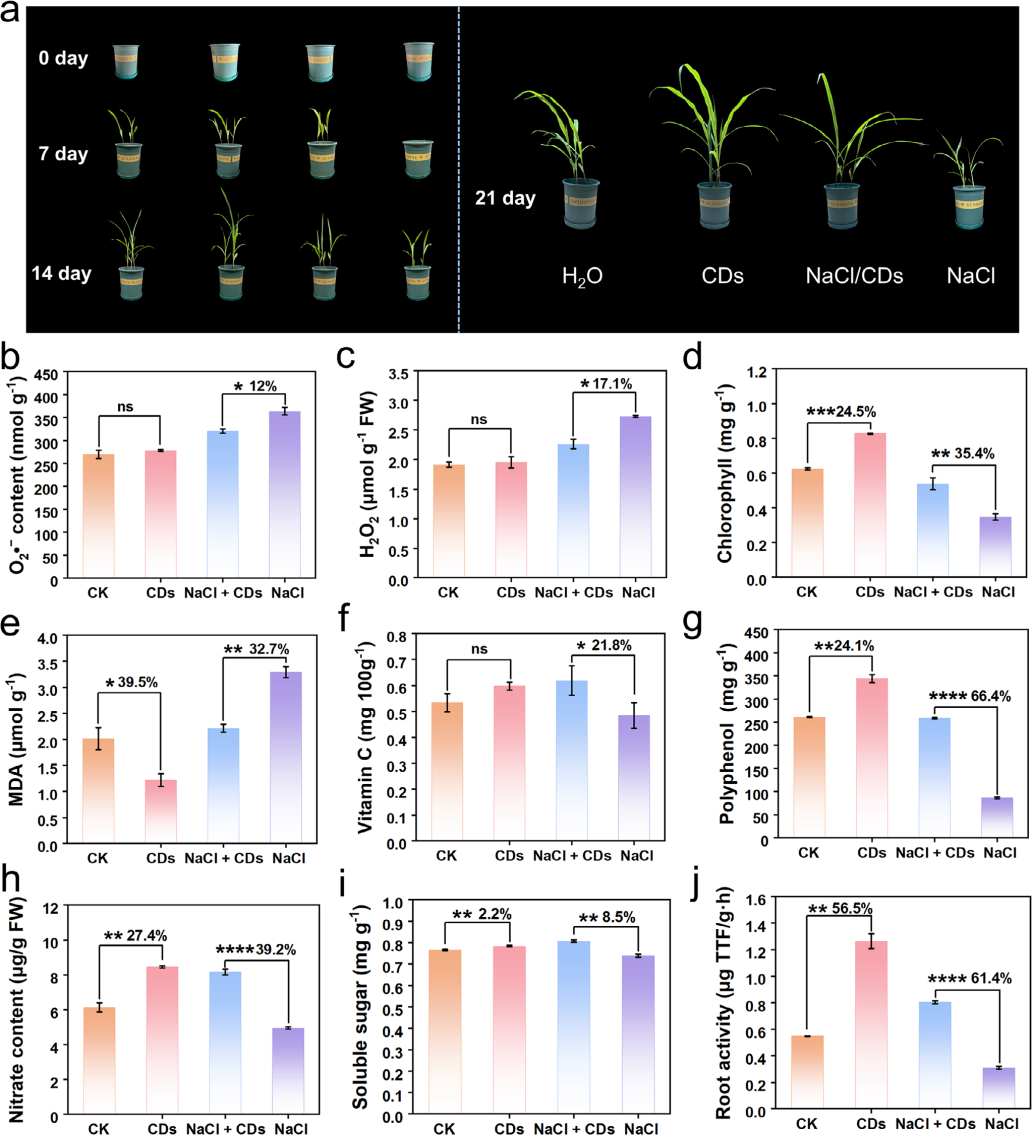
Figure 9. CD‐mediated physiological and biochemical responses of soil‐cultured maize seedlings after 21 days. a) Representative images of maize seedlings under different treatments (CK, CDs, NaCl + CDs, and NaCl) at 0, 7, 14 and 21 days. b–j) Quantitative measurements of: (b) O_2_·^−^, (c) H_2_O_2_, (d) chlorophyll, (e) MDA, (f) vitamin C, (g) total polyphenols, (h) nitrate, (i) soluble sugars, and (j) root activity. Error bars: mean ± SD (n=3). Statistical significance was determined by one‐way ANOVA with Tukey's test. *p < 0.05; **p < 0.01; ***p < 0.001; ****p < 0.0001; ns, not significant.

To better simulate saline‐alkali soil conditions, we conducted pot experiments irrigated with treatment solutions including NaCl (200 mM) or CDs (10 mg L^−1^) for the initial 7 days, followed by water maintenance (Scheme 1d). As shown in **Figures** **9**a and S10 (Supporting Information), CD significantly enhanced maize seedling growth under NaCl stress. After 21 days of growth, CDs treatment notably increased fresh weight, dry weight, root length, and shoot length by 52%, 57.3%, 37.7% and 33.7%, respectively, compared to the salt‐stressed controls (Figure S11a–d, Supporting Information). Concurrently, leaf physiological parameters also markedly improved: Tr, Pn, Gsw, and IWUE increased by 32.1%, 63.1%, 34.2%, and 47.1%, respectively (Figure S11e–h, Supporting Information). The 9.6% enhancement in Fv/Fm ratio, a key indicator of PSII photochemical efficiency,^[^ ^34^ ^]^ demonstrated CD‐mediated protection of photosynthetic apparatus under salinity (Figure S11i, Supporting Information).

To elucidate CD‐mediated stress resilience mechanisms, we analyzed antioxidant responses and metabolic profiles (Figure 9b–j). CDs treatment reduced NaCl‐induced O_2_·^−^ and H_2_O_2_ accumulation by 12% and 17.1% respectively (Figure 9b,c), while restoring chlorophyll content by 35.4% (Figure 9d). Concurrently, under salt stress, CDs application decreased lipid peroxidation (32.7% MDA reduction, Figure 9e) and enhanced antioxidant metabolites for vitamin C by 21.8% (Figure 9f) and polyphenols by 66.4% (Figure 9g). Nitrate (NO_3_^−^) plays a pivotal role in plant growth and development, and its insufficient availability is a common limiting factor for crop productivity.^[^ ^35^ ^]^ Salt stress significantly inhibited nitrate accumulation in leaves, while CDs treatment increased nitrate content by 39.2%, suggesting that CDs may improve nitrogen metabolism by enhancing root uptake capacity (Figure 9h). Soluble sugars act as osmotic regulators that enhance plant tolerance to salt stress.^[^ ^36^ ^]^ Under salt stress, CDs increased soluble sugar content by 8.5%, compared with NaCl‐treated group (Figure 9i), indicating that CDs may promote the synthesis of osmoregulatory substance, thereby maintaining cellular osmotic balance and mitigating salt‐induced dehydration. Moreover, the 61.4% recovery of root activity (Figure R5j) confirms CD‐enabled functional restoration. Taken together, these coordinated responses including ROS scavenging, osmotic adjustment, and metabolic reprogramming synergistically enhance maize salinity tolerance.

### 2.9 Transcriptomic Profiling of CD‐Induced Salt Stress Responses

**Figure 10:**
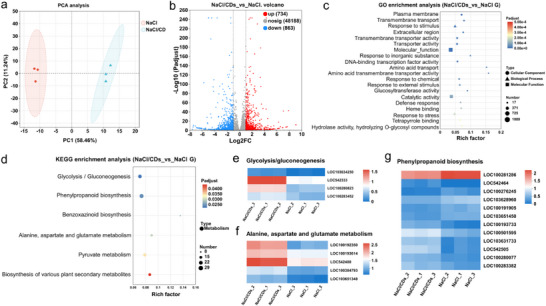
Transcriptomic analysis of CD‐induced salt tolerance mechanisms in maize. a) PCA of transcriptome profiles. b) Quantity of upregulated and downregulated DEGs (NaCl/CDs vs NaCl). c) GO enrichment analysis of DEGs in NaCl/CDs versus NaCl comparison. d) KEGG pathway enrichment analysis of DEGs. e–g) Heatmap analysis of DEGs associated with (e) glycolysis/gluconeogenesis, (f) alanine, aspartate and glutamate metabolism, and (g) phenylpropanoid biosynthesis.

To investigate the molecular basis of CDs‐mediated salt tolerance in maize, transcriptome sequencing was performed on leaf samples from NaCl‐treated maize with or without CDs. High‐quality sequencing data including 39.11 Gb clean data with over 6.28 Gb per sample, and Q30 exceeding 95.11% were obtained. Principal component analysis (PCA) and correlation analysis confirmed excellent reproducibility among the biological replicates in both the NaCl/CDs and NaCl groups (**Figure** **10**a). DESeq2 analysis identified 1597 differentially expressed genes (DEGs), including 734 upregulated and 863 downregulated genes (Figure 10b), using the screening criteria of |log2FC| ≥ 1 and padj < 0.05. These findings indicate that CDs profoundly modulated the gene expression patterns of maize under salt stress.

Gene Ontology (GO) enrichment analysis revealed significant enrichment of DEGs in functional categories including plasma membrane organization (GO:0005886), transmembrane transport (GO:0050896), defense response (GO:0006952), response to stress (GO:0006950), transmembrane transporter activity (GO:0022857), and tetrapyrrole binding (GO:0046906) (Figure 10c; Table S1, Supporting Information), revealing that CDs may exert salt tolerance effects through multiple coordinated mechanisms.

To further explore the involvement of metabolic processes, Kyoto Encyclopedia of Genes and Genomes (KEGG) enrichment analysis was performed on DEGs (Table S2, Supporting Information). The top six enriched metabolic pathways included those associated with carbohydrate metabolism (such as glycolysis/gluconeogenesis, and pyruvate metabolism), biosynthesis of other secondary metabolites (such as phenylpropanoid biosynthesis, and benzoxazine biosynthesis), and amino acid metabolism (such as alanine, aspartate, and glutamate metabolism) (Figure 10d).

In the glycolysis/gluconeogenesis pathway, NaCl/CDs group showed a 2.4‐fold increase in pyruvate kinase (PK, LOC100283452) expression and a significant 6.9‐fold increase in glyceraldehyde‐3‐phosphate dehydrogenase (GAPDH, LOC542333) expression (Figure 10e). These upregulations not only support ATP production, but also indirectly contribute to ROS scavenging by regulating NADH/NAD^+^ ratio.

In the amino acid metabolism pathway, the expression of asparagine synthase (LOC100192350) increased by 12.7‐fold, indicating enhanced synthesis of osmotic regulatory factors. Glutamine synthase (LOC542400) and glutamate synthase (LOC103651348) maintain the GS/GOGAT cycle, while glutamate dehydrogenase (LOC100193614) was upregulated by 2.4‐fold, potentially reinforcing the tricarboxylic acid (TCA) cycle and energy supply (Figure 10g).

As one of the most important secondary metabolic pathways in plants, phenylpropane biosynthesis exhibits different gene expression patterns: peroxidase genes (e.g., LOC100193733, LOC100280077) was significantly upregulated, while cinnamoyl‐CoA reductase (CCR, LOC103651450) was downregulated (Figure 10g). This regulatory mode may enhance ROS clearance ability (via peroxidase upregulation) and promote the accumulation of antioxidant compounds such as flavonoids (via CCR downregulation).

Collectively, these results suggest that CDs enhance maize salt tolerance through a multi‐tiered defense strategy: carbohydrate metabolism provides energy foundation, secondary metabolism enhances antioxidant capacity, and amino acid metabolism maintains cellular homeostasis through osmotic regulation and nitrogen balance. This comprehensive metabolic reprogramming may represent a key mechanism underlying the improved salt tolerance observed in NaCl/CDs‐treated maize.

## 3 Conclusion

In summary, we synthesized dual‐emission CD nanozymes with intrinsic SOD‐like activity via a rapid microwave method using glutathione and formamide as precursors. These CDs enhanced photosynthesis of corn through fluorescence emission that spectrally matches chlorophyll absorption profiles and promoting electron transfer efficiency. Simultaneously, CD nanozymes mitigate salt stress via SOD‐like activity and circumventing energy‐intensive stress responses. Simulated high‐salinity soils further confirmed the effectiveness of CDs in promoting salt tolerance of corn. Transcriptomic analysis revealed that CDs modulate key metabolic pathways involved in energy production, osmotic adjustment, and secondary metabolism, providing molecular evidence for their protective roles. Moreover, surface group passivation and mechanistic analysis revealed carbonyl groups and carbon core structure played essential roles in regulating both the dual‐emission properties and SOD‐like activity, providing critical insights for future functional design of CDs. This work pioneers the development of multifunctional nanomaterials that concurrently address photosynthetic limitation and oxidative damage in saline agriculture, offering a sustainable strategy to enhance crop resilience. Future studies should prioritize field validation, comprehensive environmental safety assessments, and scalable production methods to facilitate practical agricultural applications.

## 4 Experimental Section

### Materials

Glutathione, riboflavine, DL‐methionin (Met), NBT, 2‐thiobarbituric acid (TBA) 2‐methoxyphenol, trichloroacetic acid (TCA) and DCPIP were purchased from Aladdin Biochemical Technology Co., Ltd (Shanghai, China). Ethylene diaminetetraacetic acid disodium salt dihydrate (EDTA‐Na_2_) was purchased from Guangcheng Chemical reagent Co., Ltd (Tianjin, China). PS, SPI, ABTS, formamide, triethylamine and potassium persulfate (K_2_S_2_O_8_) were purchased from Maclin Biochemical Technology Co., Ltd (Shanghai, China). H_2_O_2_ (30%) and sodium hydroxide (NaOH) were purchased from Kant Chemical Co., Ltd (Laiyang, China). 1,4‐dioxane was purchased Bodi Chemical Co., Ltd (Tianjin, China). NaCl was purchased from Sinopharm Chemical Reagent Co., Ltd (Shanghai, China). All other chemicals were of analytical grade and used without further purification. All aqueous solutions were prepared with deionized water from a Milli‐Q purification system (18.2 MΩ cm, Millipore, USA).

### Preparation of CDs

GSH (0.5 g) was mixed with formamide (20 mL) and sonicated for 3 min. The mixture was transferred to microwave (100 W) oven for 3 min, and then cooled to room temperature. Then, the obtained solution was dialyzed for 24 h using dialysis bag (3500 Da). The resultant solution was prepared into powder by freeze‐drying and dissolved in water for subsequent application. The FTIR spectra were recorded on Fourier transformation infrared spectrophotometer Nicolet iS10 (Thermofisher, USA). The fluorescence spectra were measured on fluorescence spectrometer F‐7000 (Hitachi, Japan). The UV−Vis absorption spectra were measured by UV−Visible Spectrometer U‐3900 (Hitachi, Japan) and microplate reader ReadMax 1900 (Shanghai flash spectrum biotechnology Co., Ltd, China). TEM and HRTEM images were recorded by transmission electron microscope JEM 2100 F (JEOL, Japan). The XRD patterns were recorded by powder X‐Ray polycrystalline diffractometer D8ADVANCE (Bruker, Germany). The XPS spectra were recorded by using X‐ray photoelectron spectroscopy ESCALAB 250Xi (Thermo Scientific, USA). Photocurrent testing was conducted on an electrochemical workstation CHI760E (Shanghai CH Instruments Co., Ltd, China).

### The SOD‐like Activity of CDs

NBT, was a O_2_·^−^ sensitive probe. Different concentrations of CDs (0, 3, 7.5, 15, 30 µg mL^−1^), Met (13 mM), NBT (0.075 mM), riboflavin (0.02 mM), EDTA‐Na_2_ (0.01 mM) were added to the PB (10 mM, pH 7.4) under LED irradiation of 15 min. NBT reacted with the O_2_·^−^ to produce a purple‐blue product that was absorbed at 560 nm.^[^ ^37^ ^]^

### Synthesis of CDs + SPI

CDs (5 mg) and SPI (5 mg) were dispersed in carbonic acid buffer (0.1 M, pH 9.0, 5 mL) and stirred for 12 h at room temperature. The mixture was dialyzed in pure water using a dialysis bag (3500 Da) for 5–7 days.^[^ ^24^ ^]^

### Synthesis of CDs + PS

CDs (5 mg) were dissolved in water (1 mL), and then the 1,4‐dioxane (10 mL), PS (0.5 mg) and triethylamine (1 mL) were added into CDs solution. The CDs mixture was stirred at 40 °C for 24 h and the solvent was removed by rotary evaporation. The resulting product was diluted with ultrapure water, and was dialyzed with NaCl solution (0.1 M) for 24 h and pure water for 3 days using dialysis bag (3500 Da), respectively.^[^ ^24^ ^]^

### Synthesis of CDs + PS + HCl

CDs + PS (5 mg) were dissolved in NaOH (0.5 M, 5 mL) solution and stirred at room temperature for 24 h. The mixture was neutralized with HCl solution and dialyzed for 3 days.^[^ ^24^ ^]^

### Synthesis of CDs + NaBH_4_

CDs (20 mg) were dispersed in NaBH_4_ (0.5 M, 50 mL) and stirred for 24 h. The resulting solution was further neutralized with HCl and dialyzed for 3 days.^[^ ^23^ ^]^

### Synthesis of CDs + NaBH_4_ + HNO_3_

The above‐mentioned product CDs + NaBH_4_ (1 mg) was added into 0.5 M HNO_3_ (10 mL), stirred at 40 °C for 36 h, neutralized with NaHCO_3_, and dialyzed for 3 days.^[^ ^23^ ^]^

### Clearance of ABTS^·+^

ABTS^·+^ working solution was prepared by mixing 7 mM ABTS and 2.45 mM K_2_S_2_O_8_, at room temperature. The mixture was sheltered from light for 24 h and diluted with phosphate buffer (PB, 10 mM, pH 7.4) to obtain 734 nm absorbance of 0.7 ± 0.02. Different concentrations of CDs (initial concentration: 25, 50, 100, 150, 200 µg mL^−1^) were mixed with ABTS^·+^ radical stock solution and incubated at room temperature for 6 min to measure absorbance.^[^ ^38^ ^]^

### Isolation of Chloroplasts

Cleaned corn leaf tissue (10 g) was cut into small pieces and homogenized in 20 mL of sucrose buffer (0.4 M sucrose, 0.03 M Na_2_HPO_4_, and 0.02 M KH_2_PO_4_, 0.01 M KCl, pH 7.3), followed by filtration through four layers of sterile cotton gauze. Subsequently, the filtrate was centrifuged at 1000 rpm for 3 min and the supernatant was centrifuged again at 3000 rpm for 3 min.^[^ ^39^ ^]^ The precipitate was collected and redispersed in the above sucrose buffer to obtain the chloroplasts suspension. 0.1 mL of chloroplasts suspension were added to the 4.9 mL of ethanol for 30 min, and then was centrifuged again at 3000 rpm for 3 min take the supernatant. The chlorophyll concentration was measured by recording its absorbance at 652 nm in a mixed solution of ethyl alcohol, and the calculation formula was C (mg/mL) = (OD_652_ × 30) / 34.5, where C is chlorophyll concentration, and OD_652_ is chlorophyll absorption value.

### Hill Reaction

The chloroplast suspension (234.5 µg mL^−1^ chlorophyll) were mixed with DCPIP (60 µM) and CDs (0, 3, 6, 9, 12 µg mL^−1^). DCPIP was a blue dye that exhibits a powerful affinity for electrons generated by the splitting of water and can be reduced to colorless during photosynthesis.^[^ ^40^ ^]^ After the light (intensity: 4 mW·cm^−2^) irradiation for certain time (0, 1, 2, 3, 4, 5 min, respectively), the absorbance of the mix suspension at 600 nm was measured.^[^ ^22a^ ^]^

### Preparation of Chl/CDs Complex

Chloroplasts suspension was mixed with CDs in sucrose buffer for 3 h. The complex was collected by centrifuging 5000 rpm, 3 min and resuspend in sucrose buffer.

### Electrochemical Measurements

Indium tin oxide (ITO) conductive glass was used to working electrode, calomel electrode (saturated in KCl solution) and Pt were used as reference and counter electrodes. Chloroplasts (100 µL, 1.3 mg mL^−1^ chlorophyll) were mixed with CDs solution (100 µL) at varying concentrations (0, 50, and 500 µg mL^−1^). Subsequently, 100 µL aliquots of the resulting mixtures were drop‐coated onto ITO electrodes and air‐dried under ambient conditions. Nafion (20 µL, 5% w/w in water and 1‐propanol) was used to immobilize the samples. The phosphate buffered saline (pH 7.4, 0.01 M) as the electrolyte solution. Apply potential of 0.45 V.^[^ ^28^, ^41^ ^]^ The light source adopted UV light (365 nm) excitation. Turn on/off the light for 20 s as one cycle, lasting for 200 s.

### Disinfection Treatment of Seeds

The corn seeds were treated with 5% H_2_O_2_ for 30 min for disinfection, and then continuously washed with double distilled water (4–5 times). Subsequently the corn seeds were placed on absorbent paper at room temperature to dry.

### Stress Resistant Germination of Seeds

Sterilized seeds with uniform size were selected for germination. They were divided into control group (CK, H_2_O), salt stress treatment group (200 mM NaCl), salt stress treatment group with CDs (0, 1, 5, 10, and 20 mg L^−1^), respectively. The disinfectant corn seeds were placed in germinating box containing NaCl (200 mM) with or without CDs for 7 days. Germinating box (12 cm × 12 cm) was selected as the vessel, and two layers of germinating paper were laid on it. 12 seeds and the corresponding treated aqueous solution (10 mL) were added in each treatment group (ensuring full contact between the seed coat and solution), followed by incubation in a 25 °C constant‐temperature incubator under dark conditions for 7 days to monitor germination. Treatment solution (2 mL) was supplemented at 48‐h intervals throughout the experimental period.

### Corn Growth

200 uniformly sized, surface‐sterilized seeds were selected and evenly distributed on germination trays lined with 2 layers of gauze, followed by dark incubation at 25 °C for 3 days. When the roots grew to 2–3 cm, they were transferred into the planting sponge and put into the seedling tray with Hoagland nutrient solution for growth. When the seedlings grew to the stage of two leaf‐one heart, they were transferred to hydroponic culture for subsequent experiments. For photosynthesis promotion, seedlings were cultured in 1/2 Hoagland nutrient solution (500 mL) containing different concentrations of CDs (0, 1, 5, 10, and 20 mg L^−1^) for 7 days, and oxygen was supplied by oxygen pump. The solution was replaced after 4 days. To study the effects of CDs on the growth of corn seedlings under salt stress (200 mM NaCl), four treatment groups were established including CK, CDs NaCl/CDs and NaCl. The first two groups were cultured in 1/2 Hoagland nutrient solution, The latter two groups in 1/2 Hoagland nutrient solution supplemented with NaCl (200 mM). CDs (20 mg L^−1^) were administered through foliar spraying for 7 consecutive days, with a daily application of 2 mL per plant.

### Pot Experiment

The experiment included four treatment groups including H_2_O (CK), CDs, NaCl/CDs, and NaCl. The soil was mixed with treatment solutions at a 1:2 ratio, and germinated seeds with roots ≈2 cm long were transplanted into the treated soil. For the first 7 days, the corresponding treatment solutions were used for irrigation to maintain soil moisture, after which water was applied to sustain humidity. Parameters were measured after 21 days of growth.

### Transcriptomic Analysis

Transcriptome sequencing of maize leaf samples (three biological replicates per group for NaCl and NaCl/CDs treatments) was performed using the Huada T7 platform (Shanghai Majorbio Bio‐Pharm Technology Co., Ltd, China). Reference gene source: Zea_mays; Reference genome version: GCF_902167145.1; Reference genome source: https://www.ncbi.nlm.nih.gov/datasets/genome/GCF_902167145.1/; The Clean Reads of each sample were aligned with the designated reference genome, and the alignment rates ranged from 89.88% to 90.97%. A total of 40413 expressed genes were detected in this analysis, including 35021 known genes and 5392 new genes; a total of 76450 expressed transcripts, including 58359 known transcripts and 18091 new transcripts. Expression of genes and transcripts for functional database annotation analysis (NR, Swiss‐prot, Pfam, COG, GO and KEGG). The differential analysis software is: DESeq2, and the screening threshold is: |log2FC| >=1 & padjust <0.05, Under salt stress conditions, corn treated with 20 mg L^−1^ CDs served as the treatment group, while lettuce without CDs treatment was used as the control group, with three biological replicates per group.

### CLSM Imaging

Fluorescence imaging was detected by CLSM TCS SP5 (Leica, Germany). The corn was taken out and washed with distilled water at least three times. The longitudinal section of root, transverse section of stem, and leaf of the corn seedlings were analyzed using CLSM. To avoid the influence of endogenous fluorescence on the experimental results, it adjusted the parameters to decrease the auto fluorescence of plant. Under 405 nm excitation, the fluorescence images of CDs distribution in corn seedlings within the range of 630–700 nm were observed.

### Measurement of Soluble Protein Content

Coomassie brilliant blue method was used to detect soluble protein content. A standard curve was plotted with protein content on the horizontal axis and absorbance on the vertical axis. 0.2 g of fresh sample was weighted and grinded into homogenate with PB (0.05 M, pH 7.8). After mixing 1 mL of supernatant liquid with 5 mL of Coomassie Brilliant Blue G‐250 solution for 2 min, absorbance was measured at 595 nm.

### Measurement of MDA Content

MDA was determined by TBA colorimetry. Fresh leaf tissue (0.6 g) was ground into homogenate with 10% TCA (10 ml) and centrifuged at 6000 rpm for 10 min. The obtained supernatant was mixed with 0.6% TBA in 10% TCA in the same volume and incubated at 100 °C for 15 min to determine the absorbance at 450, 532, and 600 nm. The calculation formula of MDA content was as following:^[^ ^9a^ ^]^ [MDA] (µmol L^−1^) = 6.45 × (OD_532nm_ ‐ OD_600nm_) ‐ 0.56 × OD_450nm_; [MDA] (µmol/g) = [MDA] × extraction liquid volume/fresh weight.

### Measurement of Chlorophyll Content

Fresh sample (0.5 g) was put into a 50 mL centrifuge tube with 80% (v/v) acetone solution and let stand for 24 h. The absorbance at 663 and 645 nm was measured with the supernatant.^[^ ^42^ ^]^

### Measurement of Antioxidant Enzyme Activity

The aboveground part of the seedlings (0.25 g) was taken and placed into a precooled mortar contained PB (0.05 M, pH 7.8, 2 mL) and ground it into a homogeneous suspension. The homogeneous suspension was centrifuged at 10000 rpm, 4 °C for 15 min. The supernatant was collected. SOD activity was determined by NBT method. POD activity was determined by 2‐methoxyphenol method. 2‐methoxyphenol (56 µL) was dissolved in water (100 mL) at 60 °C to promote dissolution. After cooling to room temperature, 50 µL of 30% H_2_O_2_ was added and stored in the dark at 4 °C. The above solution (3 mL) was mixed with 50 µL of crude enzyme extract and the absorbance at 470 nm was measured. The change in absorbance per minute of 0.1 was defined as one enzyme activity unit.

### Determination of ROS

O_2_·^−^and H_2_O_2_ were determined using commercial assay kits (Grace Biotechnology Co., Ltd, Suzhou, China).

### Determination of Nitrate Content

Homogenized plant material (2 g) was accurately weighed (four replicates) and transferred into four graduated test tubes. Each sample was mixed with deionized water (10 mL), sealed with plastic film, and placed in a boiling water bath for 30 min for extraction. After extraction, the tubes were cooled under running tap water. The extracts were then filtered into 25 mL volumetric flasks, and the residues were repeatedly rinsed with deionized water before final volume adjustment to the mark. Next, the sample solution (0.1 mL) was pipetted into each of three test tubes, followed by the addition of 0.4 mL of 5% salicylic acid‐sulfuric acid solution. After thorough mixing, the reaction was allowed to proceed at room temperature for 20 min. Subsequently, NaOH solution (8%, 9.5 mL) was slowly added, and the mixture was shaken well. Once cooled to room temperature, the absorbance was measured at 410 nm using a blank as the reference. The nitrate‐nitrogen concentration was determined from a standard curve or calculated using a regression equation. Finally, the nitrate content in the samples was quantified based on the measured values.

### Determination of Vitamin C Content

Weigh 100 g of vegetable samples, put them into a grinder, add 100 mL of oxalic acid solution, and quickly grind them into a homogenate. Weigh 40 g of the above homogenate, add 2% oxalic acid solution to the homogenate until the volume reaches 100 mL, mix well, and filter. Take 20 mL of the above filtrate into a 250 mL conical flask, and add 3 g of activated carbon. After shaking for 60 s, filter out the activated carbon in the sample. Take the filtrate (10 mL), add thiourea solution (1%, 10 mL), and mix well. Add the above solution (4 mL) to 3 test tubes. One of the test tubes was marked as a blank control, and the other two were respectively added with 1.0 mL of 2% 2,4‐dinitrophenylhydrazine solution. Place the above 3 test tubes in a water bath (≈37 °C) for a reaction of ≈3 h. When the samples in the test tubes reach room temperature, add 1.0 mL of 2% 2,4‐dinitrophenylhydrazine solution to each of the three test tubes. Then transfer the test tubes to an ice bath condition, and respectively add 85% sulfuric acid (5 mL) to each. The dropping process should be slow, and the time should be no less than 60 s. Place each test tube at room temperature for color development for 30 min. Measure the absorbance values of each sample at a wavelength of 523 nm.

### Determination of Root Activity

Weigh 0.5 g of root tip samples, sequentially add 2,3,5‐triphenyltetrazolium chloride (TTC) solution (5 mL, 0.4%) and PB (5 mL, 1/15 M), mix thoroughly, and ensure the root tip segments were completely immersed in the reaction solution. Incubate in a 37 °C constant‐temperature incubator under dark conditions for 2 h to allow color development in the root tip segments. Transfer the colored root tip segments to a stoppered graduated test tube, add methanol (10 mL) to completely immerse the segments, then place the test tube in an incubator at 30–40 °C for ≈2 h until the root tip segments turn completely white. The absorbance at 485 nm was recorded.

### Determination of Polyphenol Content

Place fresh sample (0.1 g) in a 50‐mL centrifuge tube. Add 95% ethanol (6 mL), and perform ultrasonic extraction in a water bath at 50 °C for 30 min. Transfer the supernatant to a 25‐mL volumetric flask. Repeat the extraction process three times. Combine the extracts, dilute to the mark with 95% ethanol, and shake well to obtain the sample solution. Pipette 1.0 mL of the sample solution into a 10‐mL volumetric flask. Sequentially add 5 mL of Folin‐Ciocalteu's phenol reagent diluted 10 times. After stand for 30 s, add 7.5% sodium carbonate solution (2 mL), dilute to volume with distilled water, and shake thoroughly. Keep the solution at 30 °C in the dark for 1 h, and then measure the absorbance at a wavelength of 760 nm.

### Determination of Photosynthetic Indexes

The Pn, Tr, Gsw and IWUE of maize leaves were measured using the LI‐6800 portable photosynthesis system (LI‐COR, USA). For each measurement, fully expanded leaves were selected and measured three times. The ETR of leaves was measured by a Chlorophyll fluorescence imaging system IMAGING‐PAM (WALZ, Germany), with the leaves being dark‐adapted for 30 min prior to the test.

### Statistical Analysis

The data were expressed as the means ± standard deviation (SD) with at least three replicate experiments. Significant difference between groups was determined with one‐way analysis of variance (ANOVA) followed by Tukey's post‐hoc test for multiple comparisons using Graphpad Prism 10 software. Statistical significance: *p < 0.05, significant; **p < 0.01, ***p < 0.001 and ****p < 0.0001, highly significant. *P* > 0.05, ns, not significant.

## Conflict of Interest

The authors declare no conflict of interest.

## Author Contributions

C.S, X.P., and J.T. contributed equally to this work. L.H. and X.X. conceived and supervised the study. X.P., J.T., C.S., and Y.C. performed the experiments, and visualized the results. C.S. also provided plant‐related instrumental support. All authors analyzed the results. L.H. wrote the original manuscript and led the extensive revision with critical input from X.P., X.X., J.T. and all the other co‐authors.

## Supporting information



Supporting Information

## Data Availability

The data that support the findings of this study are available from the corresponding author upon reasonable request.
