# Supplementary material for: Dual‐Emission Carbon Dot Nanozymes Mitigate Salt Stress and Enhance Photosynthesis for Crop Germination and Growth
Source: Adv Sci (Weinh). 2025 Sep 29;12(46):e06906. doi: 10.1002/advs.202506906 (PMC12697893; doi:10.1002/advs.202506906)
Supplement: Supplementary file 1 — Supporting Information [file ADVS-12-e06906-s001.pdf]

Supporting Information

Dual-Emission Carbon Dot Nanozymes Mitigate Salt Stress and Enhance Photosynthesis for Crop Germination and Growth

Chengfu Su[#], Xin Peng[#], Jingying Tan[#], Yujie Chang, Naifei Pan, Haiying Chen, Xiaokai Xu, Lei Han**

[#] C. Su, X. Peng, and J. Tan contributed equally to this work.

**E-mails: hanlei@qau.edu.cn (L. Han); xxk@qau.edu.cn (X. Xu)*

Contents

Figure S1. The powder photograph of carbon dots (CDs) S3

Figure S2. Fluorescence spectra of modified CDs + PS, CDs + $NaBH_4$ and CDs + SPI CDs at 355–435 nm excitation S4

Figure S3. Reduction rates of DCPIP mediated by Chl/CDs complexes with varying concentrations of CDs (0–12 μg/mL) under 3 min light illumination (4 mW cm^{-2}) S5

Figure S4. IWUE of CDs-treated corn seedlings after one week of hydroponic culture S6

Figure S5. CLSM images of corn seedlings treated with water for 7 days under 405 nm excitation S7

Figure S6. The clearance rate of $ABTS^{\cdot+}$ by CDs at different concentrations S8

Figure S7. Hydrodynamic size distribution and zeta potential diagrams of CDs in various solutions. S9

Figure S8. Quantitative analysis of the activity of a) SOD and b) POD in corn seeds under salt stress with and without CDs treatment (20 mg/L) S10

Figure S9. CDs-mediated protection of corn seeds under salt stress S11

Figure S10. Phenotypic comparison of maize seedlings under soil cultivation. Treatments S12

Figure S11. Growth and photosynthetic parameters of soil-cultivated maize seedlings under salt stress at 21 days S13

Table S1. GO enrichment analysis of DEGs between NaCl + CDs and NaCl treatments S14

Table S2. KEGG pathway enrichment of DEGs in NaCl/CDs vs. NaCl comparison.. S15

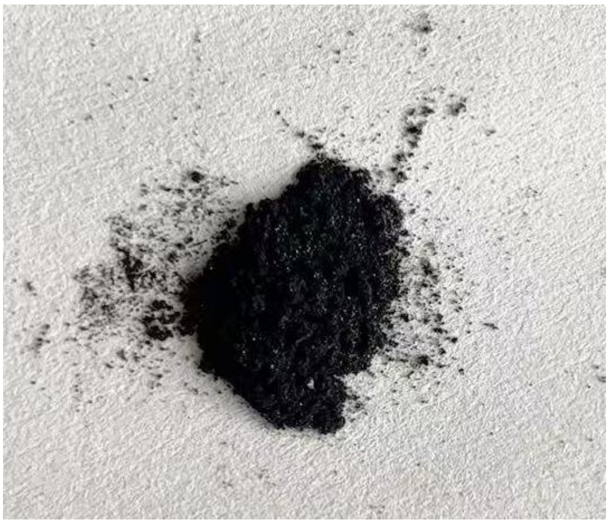

Figure S1. The powder photograph of carbon dots (CDs).

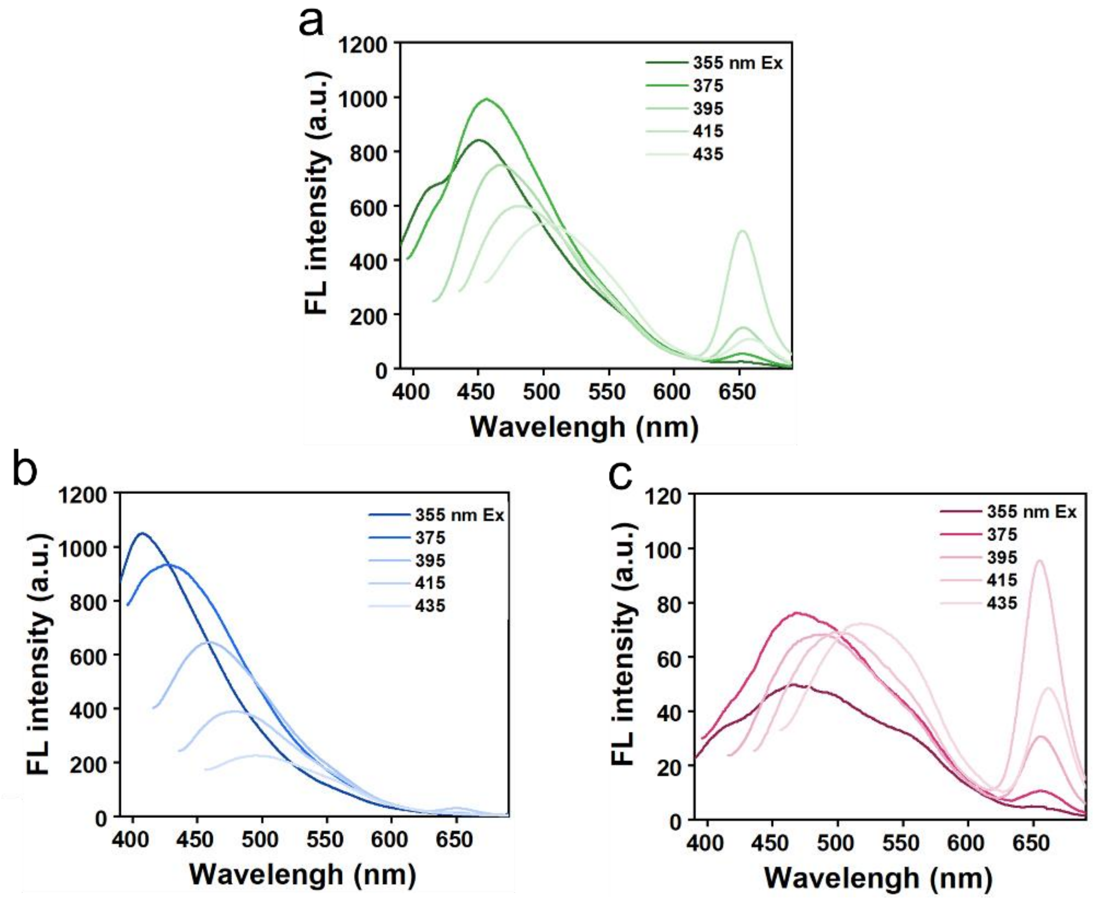

Figure S2. Fluorescence spectra of modified a) CDs + PS, b) CDs + $NaBH_4$ and c) CDs + SPI CDs at 355–435 nm excitation.

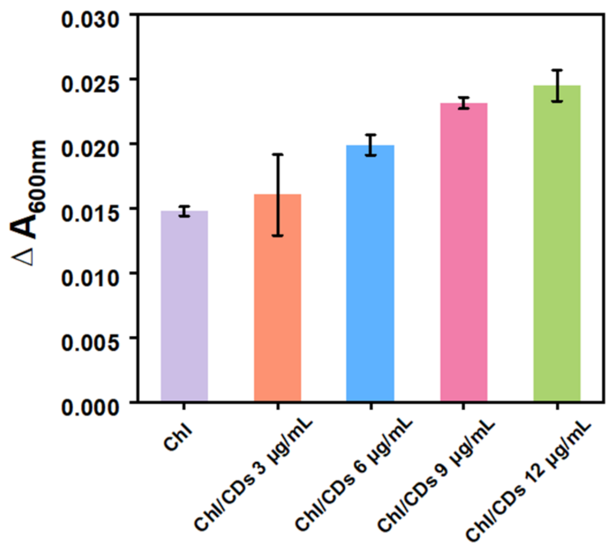

Figure S3. Reduction rates of DCPIP mediated by Chl/CDs complexes with varying concentrations of CDs (0–12 μg/mL) under 3 min the light illumination (4 mW cm^{-2}). Error bars: mean ± SD (n=3).

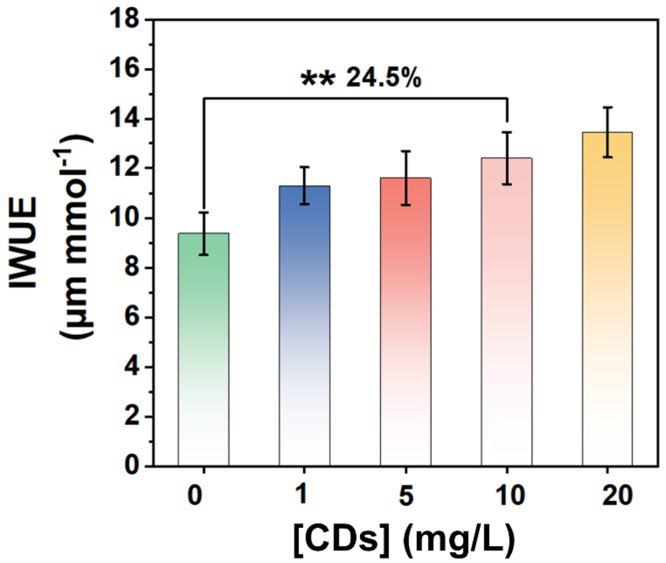

Figure S4. IWUE of CDs-treated corn seedlings after one week of hydroponic culture. Error bars: mean ± SD (n=3). Statistical significance was determined by one-way ANOVA with Tukey's test. $^{**}P < 0.01$.

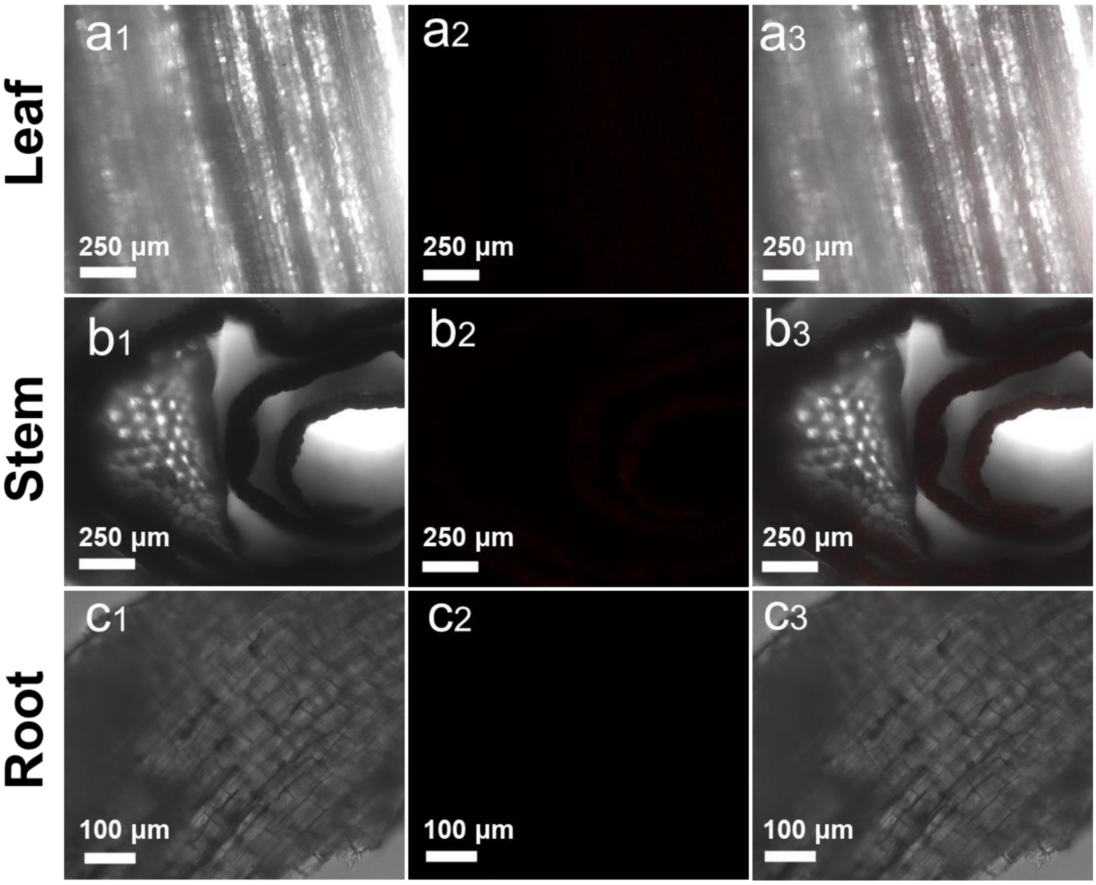

Figure S5. CLSM images of corn seedlings treated with water for 7 days under 405 nm excitation. The data of leaf tissue (a), cross section of stem (b), and longitudinal section of root (c) were collected in the range of the bright field image in (1), red emission image (630−700 nm) in (2), and the overlay of the fluorescence microscopy image and bright field image in (3).

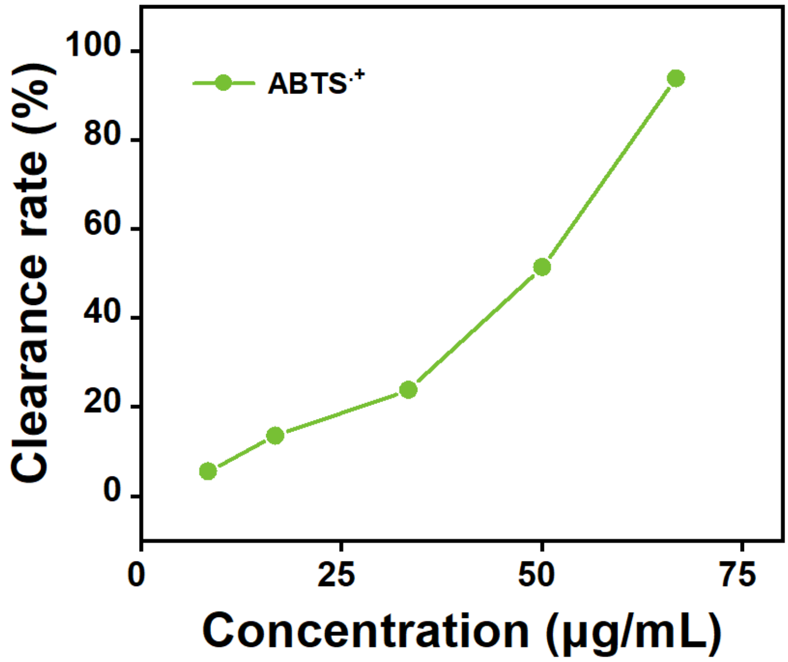

Figure S6. The clearance rate of ABTS·$^+$ by CDs at different concentrations.

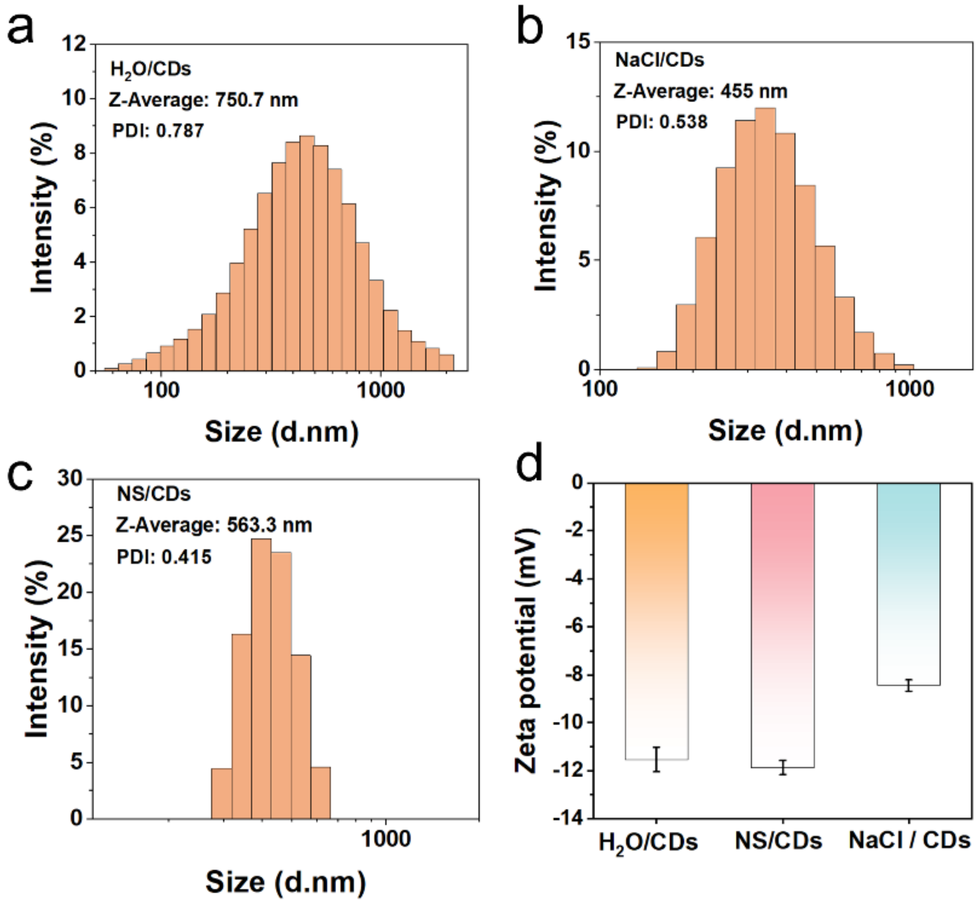

Figure S7. Hydrodynamic size distribution and zeta potential diagrams of CDs in various solutions. a-c) Hydrodynamic size distribution diagrams of CDs in H_2O (H_2O/CDs), NaCl solution (NaCl/CDs), and 1/2 Hoagland nutrient solution (NS/CDs). d) Zeta potential diagrams of CDs in these solutions. Error bars: mean ± SD (n=3).

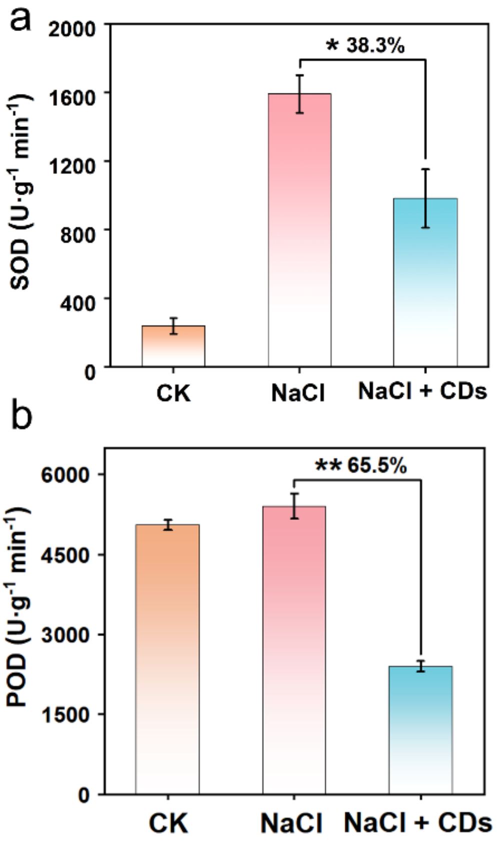

Figure S8. Quantitative analysis of the activity of a) SOD and b) POD in corn seeds under salt stress with and without CDs treatment (20 mg/L). Error bars: mean ± SD (n=3). Statistical significance was determined by one-way ANOVA with Tukey's test. *$P < 0.05$, significant; **$P < 0.01$, highly significant; $P > 0.05$, ns, not significant.

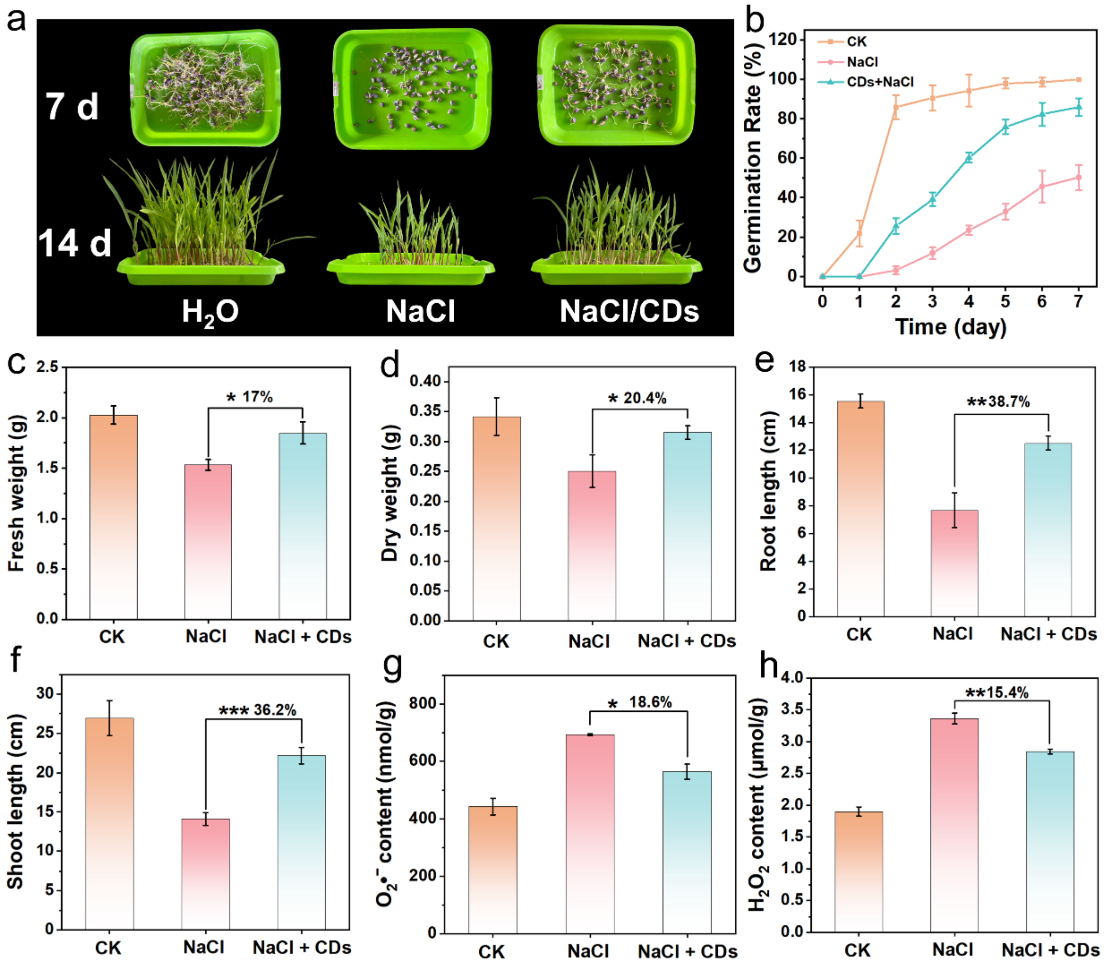

Figure S9. CDs-mediated protection of corn seeds under salt stress. a) Germination phenotypes with CDs treatments (20 mg/L) under NaCl stress (200 mM) for 7 and 14 days. b) Seed germination rate for 14 days. Quantitative analysis of c) fresh weight, d) dry weight, e) root length, f) stem length, g) superoxide anion content, h) hydrogen peroxide content in corn seeds under salt stress with and without CDs treatment (20 mg/L) after 14 days. Error bars: mean ± SD (n=3). Statistical significance was determined by one-way ANOVA with Tukey's test. **P* < 0.05, significant; ***P* < 0.01, and ****P* < 0.001, highly significant; *P* > 0.05, ns, not significant.

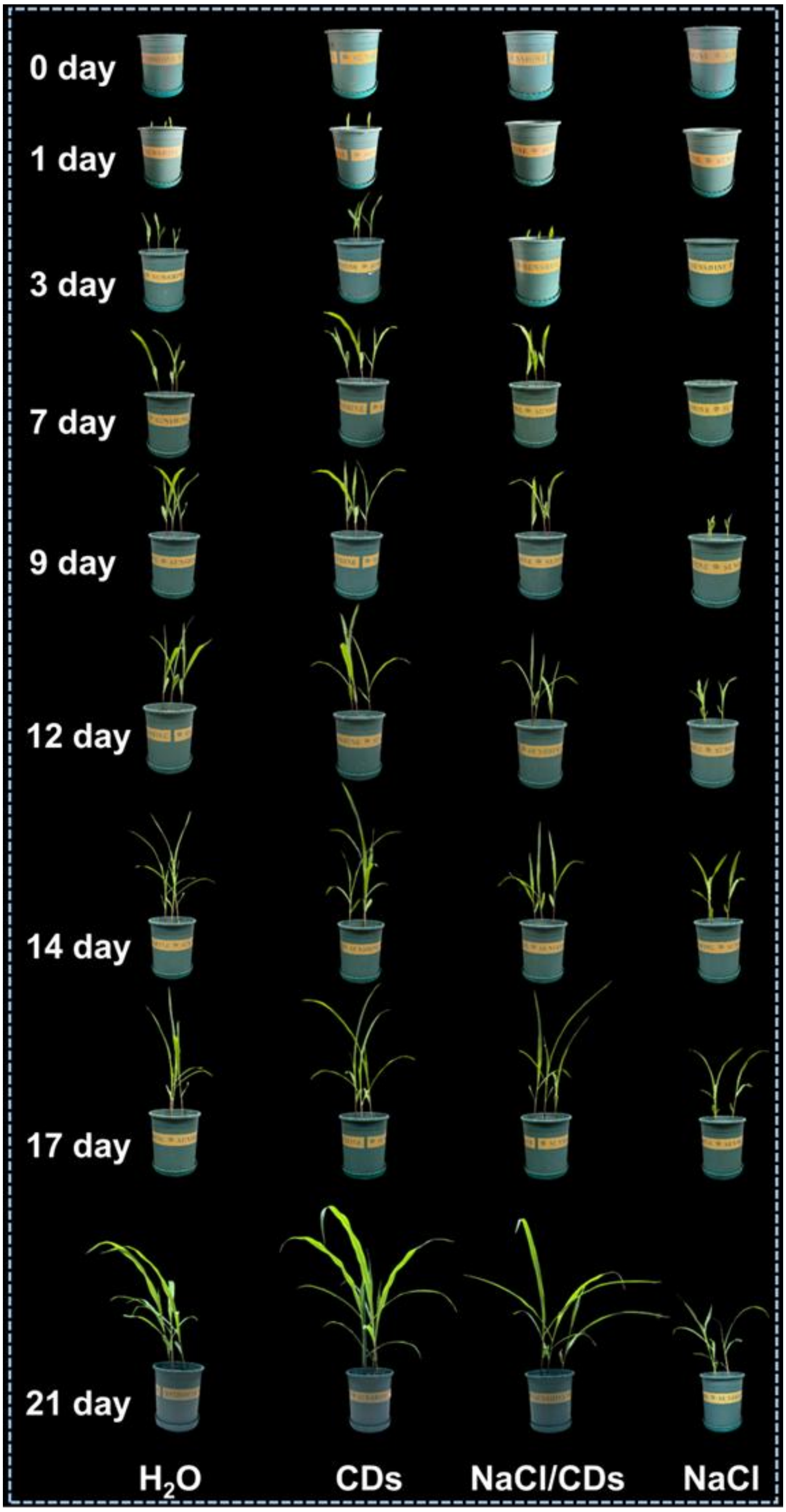

Figure S10. Phenotypic comparison of maize seedlings under soil cultivation. Treatments: control (H_2O), CDs (10 mg/L), NaCl (200 mM), and NaCl+CDs.

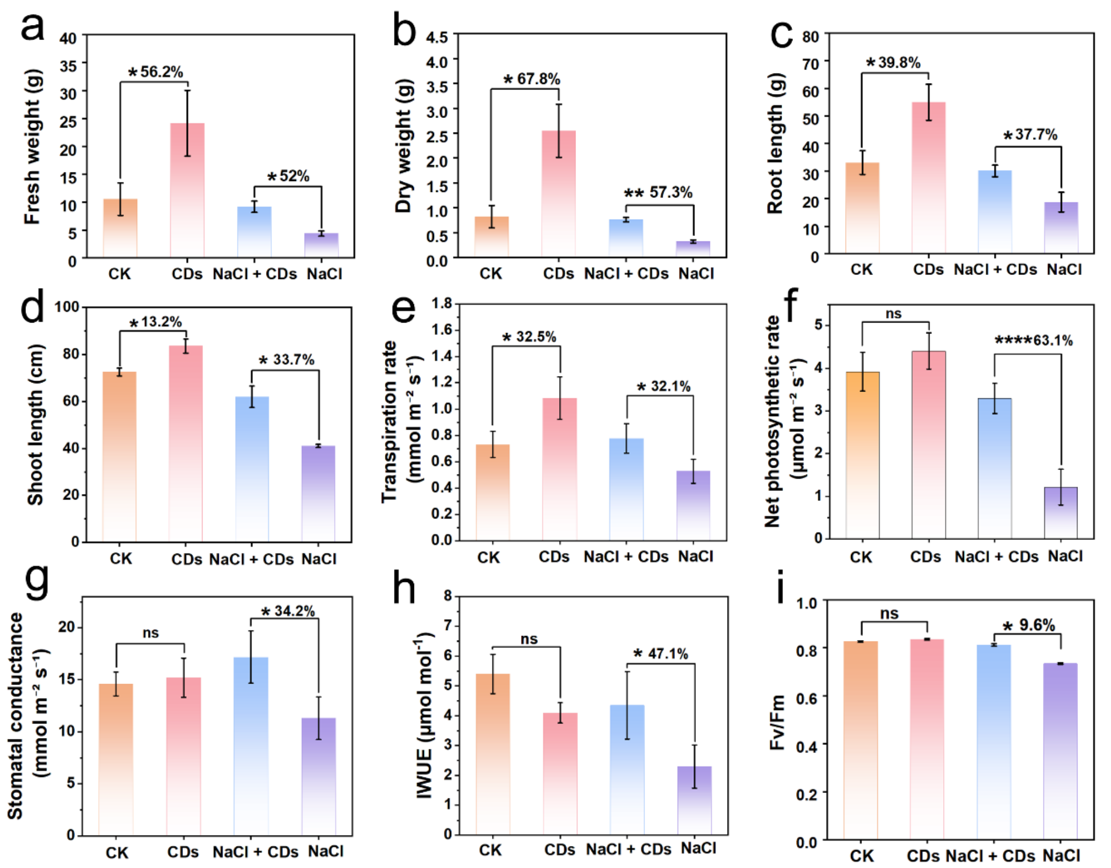

Figure S11. Growth and photosynthetic parameters of soil-cultivated maize seedlings under salt stress at 21 days. a) Fresh weight, b) dry weight, c) root length, d) shoot length, e) transpiration rate, f) net photosynthetic rate, g) stomatal conductance, h) instantaneous water use efficiency, i) Fv/Fm in the CK, CDs, NaCl/CDs and NaCl groups. Error bars: mean ± SD (n=3). Statistical significance was determined by one-way ANOVA with Tukey's test. *$P < 0.05$, significant; **$P < 0.01$, ****$P < 0.0001$, highly significant; ns, not significant.

Table S1. GO enrichment analysis of DEGs between NaCl + CDs and NaCl treatments

Category	Description	GO ID
Biological process	transmembrane transport	GO:0055085
	response to stimulus	GO:0050896
	response to inorganic substance	GO:0010035
	amino acid transport	GO:0006865
	response to chemical	GO:0042221
	response to external stimulus	GO:0009605
	defense response	GO:0006952
	response to stress	GO:0006950
Cellular component	plasma membrane	GO:0005886
	extracellular region	GO:0005576
Molecular function	transmembrane transporter activity	GO:0022857
	transporter activity	GO:0005215
	molecular_function	GO:0003674
	DNA-binding transcription factor activity	GO:0003700
	amino acid transmembrane transporter activity	GO:0015171
	glucosyltransferase activity	GO:0046527
	catalytic activity	GO:0003824
	tetrapyrrole binding	GO:0046906
	hydrolase activity, hydrolyzing O-glycosyl compounds	GO:0004553

Table S2. KEGG pathway enrichment of DEGs in NaCl/CDs vs. NaCl comparison

Gene Name	Gene Description	KO ID
LOC100191418	putative O-Glycosyl hydrolase superfamily protein	K15920
LOC100191460	phosphoenolpyruvate carboxylase3	K01595
LOC100191905	Peroxidase 64, transcript variant X1	K00430
LOC100192350	asparagine synthetase 3	K01953
LOC100192351	asparagine synthetase 4	K01953
LOC100193614	glutamic dehydrogenase2	K00261
LOC100193733	peroxidase 12	K00430
LOC100273555	UDP-glycosyltransferase 75B1	K21371
LOC100276245	putative class III secretory plant peroxidase family protein	K00430
LOC100277344	benzoxazinone synthesis 8	K13227
LOC100280077	Peroxidase 59	K00430
LOC100280449	Pyrophosphate--fructose 6-phosphate 1-phosphotransferase subunit alpha 2	K00895
LOC100280823	triosephosphate isomerase, cytosolic	K01803
LOC100281060	amidophosphoribosyltransferase	K00764
LOC100281286	peroxidase 45	K00430
LOC100281699	cytokinin-O-glucosyltransferase 2	K21374
LOC100282748	aldehyde dehydrogenase family 7 member A1	K14085
LOC100282831	1-aminocyclopropane-1-carboxylate oxidase	K13229
LOC100283171	aldose 1-epimerase	K01785
LOC100283382	peroxidase 39, transcript variant X1	K00430
LOC100283452	pyruvate kinase, cytosolic isozyme	K00873
LOC100283617	dihydrolipoyllysine-residue acetyltransferase component of pyruvatedehydrogenase complex	K09699
LOC100284047	succinate semialdehyde dehydrogenase	K17761
LOC100285433	AER	------
LOC100285796	dihydrolipoyllysine-residue acetyltransferase component of pyruvatedehydrogenase complex	K00627
LOC100382182	ppi-phosphofructokinase	K00895
LOC100382449	Aldehyde dehydrogenase, transcript variant X1	K00128
LOC100384793	alanine aminotransferase 2	K00814
LOC101202696	acetyl-CoA acetyltransferase, cytosolic 1	K00626
LOC103628763	UDP-glycosyltransferase 82A1	------
LOC103628960	peroxidase 47	K00430
LOC103631733	peroxidase 44	K00430
LOC103639152	tryptamine hydroxycinnamoyltransferase 2	------
LOC103649667	cyanidin 3-O-rutinoside 5-O-glucosyltransferase-like	K21371

LOC103650610	4-hydroxy-7-methoxy-3-oxo-3,4-dihydro-2H-1,4-benzoxazin-2-yl glucoside beta-D-glucosidase 2, chloroplastic-like	K01188
LOC103651348	glutamate synthase 1 [NADH], chloroplastic, transcript variant X1	K00264
LOC103651450	cinnamoyl-CoA reductase-like SNL6	K09753
LOC103655673	acetyl-CoA carboxylase 1-like	K11262
LOC107282093	Caffeoyl-CoA O-methyltransferase1	K00588
LOC109943052	1-aminocyclopropane-1-carboxylate oxidase homolog 7, transcript variant X1	K13229
LOC541914	aldehyde dehydrogenase 5	K12355
LOC542117	benzoxazinless 1	K13222
LOC542333	cytosolic glyceroldehyde-3-phosphate dehydrogenase GAPC3	K00134
LOC542393	phosphoenolpyruvate carboxylase 4	K01595
LOC542400	glutamine synthetase 2	K01915
LOC542414	beta glucosidase 1	K01188
LOC542464	anionic peroxidase	K00430
LOC542479	phosphoenolpyruvate carboxylase 7	K01595
LOC542505	peroxidase 3	K00430
LOC542567	aldehyde dehydrogenase 2	K00128
LOC542651	pyruvate decarboxylase	K01568
LOC542717	isoflavone reductase-like 1	K23050; K21568
